# A model of working memory for encoding multiple items and ordered sequences exploiting the theta-gamma code

**DOI:** 10.1007/s11571-022-09836-9

**Published:** 2022-07-16

**Authors:** Mauro Ursino, Nicole Cesaretti, Gabriele Pirazzini

**Affiliations:** Department of Electrical, Electronic and Information Engineering “Guglielmo Marconi”, University of Bologna, Campus of Cesena Area di Campus Cesena Via Dell’Università 50, 47521 Cesena, FC Italy

**Keywords:** Working memory, Brain rhythms, Neural mass models, Theta-gamma phase coupling, Binding and segmentation, Spatio-temporal memory

## Abstract

Recent experimental evidence suggests that oscillatory activity plays a pivotal role in the maintenance of information in working memory, both in rodents and humans. In particular, cross-frequency coupling between theta and gamma oscillations has been suggested as a core mechanism for multi-item memory. The aim of this work is to present an original neural network model, based on oscillating neural masses, to investigate mechanisms at the basis of working memory in different conditions. We show that this model, with different synapse values, can be used to address different problems, such as the reconstruction of an item from partial information, the maintenance of multiple items simultaneously in memory, without any sequential order, and the reconstruction of an ordered sequence starting from an initial cue. The model consists of four interconnected layers; synapses are trained using Hebbian and anti-Hebbian mechanisms, in order to synchronize features in the same items, and desynchronize features in different items. Simulations show that the trained network is able to desynchronize up to nine items without a fixed order using the gamma rhythm. Moreover, the network can replicate a sequence of items using a gamma rhythm nested inside a theta rhythm. The reduction in some parameters, mainly concerning the strength of GABAergic synapses, induce memory alterations which mimic neurological deficits. Finally, the network, isolated from the external environment (“imagination phase”) and stimulated with high uniform noise, can randomly recover sequences previously learned, and link them together by exploiting the similarity among items.

## Introduction

Brain rhythms are large fluctuations in neuronal activity, which are observed with scalp EEG and local field potential recordings, and reflect the synchronized activity of large populations of neurons (Buzsáki and Draguhn 2004). This synchronized activity plays a relevant role in cognition, since the execution of complex cognitive tasks requires the co-ordinate participation of multiple neural populations, which form a functional network and work in synergy (Ward et al. 2003; Wang 2010; Fries 2015). Synchronization of neural activity is also relevant for memory consolidation and learning, favoring optimal synapse reinforcement among neurons which fire together, and depotentiation of synapses among neurons that do not show any evident temporal correlation (Düzel et al. 2010). The relevant role of brain rhythms in cognition is further emphasized by the enormous amount of observations in humans and animals, showing that the shape, power and phase coupling of brain rhythms is altered in different tasks, that these patterns are modulated by sleep and attention, and reflect various aspects of human perception, memory and behavior (Wang 2010; Roux and Uhlhaas 2014; Watson and Buzsáki 2015).

Among the different oscillations in brain activity, a pivotal role in recent years is ascribed to the theta (4–8 Hz) and gamma (> 30 Hz) rhythms, especially during memory tasks which require storage and recall of multiple items.

Briefly, for memory to correctly function, the maintenance, binding and ordering of perceptual information within neuronal networks are fundamental. Hence, a neural code that specifies how different elements of the same item are joined together (*binding problem*) and separated from elements of other items (*segmentation problem*) is required. Moreover, since time is an essential element of declarative memory, a code to order these items in a correct temporal sequence is also needed.

An influential hypothesis is that the previous problems are dealt with using the so-called theta-gamma code (Lisman and Jensen 2013). According to this coding scheme, a subset of cells that fire in phase (i.e., synchronized) during a gamma cycle would represent aspects of a same item (they are often referred to as a *cell assembly*); conversely, cells that fire at different gamma cycles would represent aspects of different items. In this way, multiple items can be maintained in memory simultaneously using a sort of time division. A temporal order for the different items may be realized with the support of the theta-band activity: neurons representing different items are active at different instants during the excitatory period of the theta cycle (on period), with the phase representing information on their temporal arrangement. The same sequence can then be repeated again and again, at each theta period. Since the ratio between the theta and gamma periods is approximately between 6 and 8, this poses an upper bound on the number of items maintained in temporal order (Lisman and Jensen 2013).

Indeed, many experiments have investigated the role of theta/gamma oscillations in past years, especially in rodents, revealing that higher frequency gamma oscillations (40 Hz) are often nested within slower theta oscillations, fire at specific phases of the theta cycle (Soltesz and Deschênes 1993; Bragin et al. 1995; Belluscio et al. 2012; Colgin 2016) and exhibit a precession phenomenon (Skaggs et al. 1996; Tsodyks et al. 1996; O’Keefe and Burgess 2005). More recently, the study of theta-gamma coupling has been performed in humans too, and now represents a fundamental aspect in cognitive neuroscience (Canolty et al. 2006; Sauseng et al. 2010, 2019; Chaieb et al. 2015; Heusser et al. 2016).

While many results definitely demonstrate the presence of this theta-gamma code in the hippocampus, where it is especially involved in episodic and spatial memory (Lisman 2005; Colgin 2016), more recent results suggest that this mechanisms also occur in various other parts of the cortex (especially in fronto-temporal regions in humans) where it can also be involved in semantic memory and working memory (Canolty et al. 2006; Axmacher et al. 2010; Roux and Uhlhaas 2014; Alekseichuk et al. 2016; Heusser et al. 2016; Bahramisharif et al. 2018; Köster et al. 2018; Vivekananda et al. 2021). A recent idea is that the hippocampus can work in concert with fronto-temporal regions, to realize relational networks useful for sematic associations (Eichenbaum 2004) and to estimate current time passing (Sakata 2006).

Furthermore, the mechanisms for brain rhythm generation can be altered in some neurological disorders, and these changes may contribute to some of the characteristics of the pathology (Buzsáki and Watson 2012). In particular, various studies suggest that schizophrenia is associated with a variety of abnormalities in gamma and theta oscillations (Spencer et al. 2003; Spencer 2008; Kirihara et al. 2012; Senkowski and Gallinat 2015), and that theta-gamma coupling is significantly and selectively impaired during working memory tasks in schizophrenic patients compared to healthy controls (Barr et al. 2017). Furthermore, communication between brain areas as well as their oscillatory activity can be altered in epilepsia (Froriep et al. 2012; Inostroza et al. 2013; Laurent et al. 2015; Kitchigina 2018). In humans with Alzheimer disease, an enhanced frequency coupling was revealed between the gamma and theta bands compared to healthy control (Wang et al. 2017), while altered theta-gamma coupling was associated with working memory deficits (Goodman et al. 2018; Kitchigina 2018).

Finally, but not less important, theta-gamma rhythms play a role in memory consolidation during sleep (Boyce et al. 2016; Bandarabadi et al. 2019), and can contribute to inference reasoning and imagination of new concepts beyond direct experience (Gupta et al. 2010; Backus et al. 2016).

All previous considerations emphasize the enormous impact that knowledge on gamma-theta brain rhythms can have for neuroscience, in many different cognitive domains. However, despite the huge increase in experimental and clinical studies on the subject in recent years, a systematic comprehension of the mechanisms sub-serving rhythm cooperation in the brain, and of their role in cognition, is still lacking, especially for what concerns information processing and integration in large brain networks. Actually, the enormous amount of data and results risk being insufficiently understood, if not summarized into a more theoretical vision. In this regard, neurocomputational models, inspired by biology, can play a relevant role to condense information, to provide quantitative methods to check new ideas and hypotheses, to suggest testable predictions for driving experiments and, more generally, as a powerful repository of our knowledge in complex domains where qualitative reasoning in often inadequate.

Indeed, several models of theta-gamma coupling have been proposed in the past decades, emphasizing the sequential organization of memory, especially with reference to the hippocampus.

Two pioneering models are those by Levy, and by Jensen and Linsman.

Levy (1989, 1996) proposed a model of the hippocampal area CA3, in which a sparse recurrent connectivity combined with rapid synaptic plasticity can produce asymmetric connections, able to represent sequences of information. Jensen and Lisman, in a series of papers (Lisman and Idiart 1995; Jensen and Lisman 1996, 2005) proposed a more complex recurrent model of the hippocampus, including the dentate gyrus, CA3 and CA1; their model is able to store multiple items via Long Term Potentiation (LTP), thus realizing a temporal multiplexing mechanism that is clocked by the theta and gamma oscillations.

Other studies (Wallenstein and Hasselmo 1997; Cutsuridis and Hasselmo 2012) are based on sophisticate multicompartmental models of pyramidal neurons and local inhibitory interneurons, laying particular emphasis on the function of GABAergic mechanisms and on the role of different excitatory and inhibitory hippocampal cell types. With a same aim, Mysin et al. (2019) recently presented a model which includes a detailed biophysical representation of major cell types related to the theta rhythm emergence: excitatory pyramidal cells and two types of inhibitory interneurons.

Other models are more oriented to mathematical analysis: in the standard leaky integrate-and-fire model by Chance (2012), CA1 pyramidal cells are driven by dual input components arising from CA3 and from layer III of entorhinal cortex; the author studied how manipulations in these areas can affect the theta-phase of CA1 place field spikes. Using a minimal computational model, Jaramillo and Kempter (2017) modelled how phase precession of place cells and of grid cells in the hippocampal formation can be inherited by the prefrontal cortex and the ventral striatum. Thurley et al. (2008). mathematically investigated how phase precession can be produced by a threshold model, which uses synaptic facilitation in combination with oscillations of the membrane potential.

Finally, we can mention some other models able to simulate not only phase precession during spatial navigation, but also the phenomena of replay and pre-play, i.e., an offline sequential activity of place cells not driven by any external stimulus. Buzsáki (1989) used a moving threshold model, in which neurons receive a subthreshold activation at rest, and threshold is lowered gradually. This model predicted the existence of replay in both forward and reverse order. The model by Hopfield (2010) is based on continuous attractor dynamics, which can produce exploration of trajectories never experienced before. Dockendorf and Srinivasa (2013) recently presented a recurrent model using spiking neurons, for learning new spatiotemporal patterns.

All previous models, however, have some limitations. Most of them are devoted to investigation of place cell behavior and focus their attention only on individual neurons in the hippocampus and on the problem of memorization and retrieval of a spatial task. Our previous summary, however, clearly suggests that theta-gamma coupling may play a relevant role in many other cognitive domains (including semantic memory and working memory) and may encompass other areas beside the hippocampus, such as frontal, temporal and perhaps parietal regions (Canolty et al. 2006; Colgin 2011). Hence, we need more general models too, which can simulate rhythm coupling and memory formation in a larger cognitive scenario, exploiting the theta-gamma interaction in a wide assembly of neural populations. In particular, in many complex cognitive problems, integration of neural information exploits a “distributed code”, involving large populations of neurons which fire together to represent different aspects of memory.

An important class of models, useful to study interactions among neural populations instead of individual neurons, is represented by Neural Mass Models (NMMs). In these models, the output of a computational unit describes the activity of groups of neurons, which share the same characteristics and exhibit a similar collective behavior. This choice reduces the number of parameters and state variables, allows better generalization, and facilitates the analysis of results compared with more detailed models based on spiking neurons.

Recently, we developed a model for the study of theta-gamma coupling, exploiting the dynamics of neural masses (Cona et al. 2012; Cona and Ursino 2013). The model was able to store and reproduce sequence of events using Hebbian and anti-Hebbian learning paradigms. A subsequent version (Cona and Ursino 2015) trained to reproduce place cells behavior and rat’s navigation in a maze, simulated not only phase precession, but also replay, reverse replay and imagination of new paths in the offline sequential activity.

The aim of the present work is to significantly improve the previous multi-layer neural mass model to investigate the role of theta and gamma rhythms in working memory. The model aspires to summarize different aspects of working memory into a single theoretical framework. In particular, we will analyze the following problems: (i) how a sequence of objects can be memorized in a network and recovered in working memory, starting from an incomplete cue of the first object only, by exploiting a theta-gamma code. We call this “*the sequence ordering working memory*”, since the major aim is to remember items in a correct order; (ii) how different objects, previously learned, can be simultaneously recovered in working memory, without any specific order (that is, a segmentation problem) starting from partial cues of each object provided together. We call this “*the semantic working memory*”, since its aim is to maintain task-relevant conceptual knowledge; (iii) to provide a preliminary analysis on the role of some parameter changes, simulating possible pathological disorders (such as Alzheimer Disease or Schizophrenia); (iv) to investigate network behavior in an “imagination” or “dreaming” condition, when working memory is free to recover previously stored information without an external cue.

The present model wishes to represent a general framework on the possible role of theta-gamma oscillations in working memory, with potential application for different brain areas. However, specific comparison with neurophysiological data are given with reference to the interactions prefrontal cortex-hippocampus, which are highly documented in the literature and are known to play a relevant role in memory (Eichenbaum 2017). The final discussion focuses on the main virtues and limitations of the model, and provides key elements for further improvements and for future studies.

## Method

In this section we present a qualitative description of the model. A complete quantitative description, with all equations and parameter’s numerical values, is given in the Appendix.

**Fig. 1:**
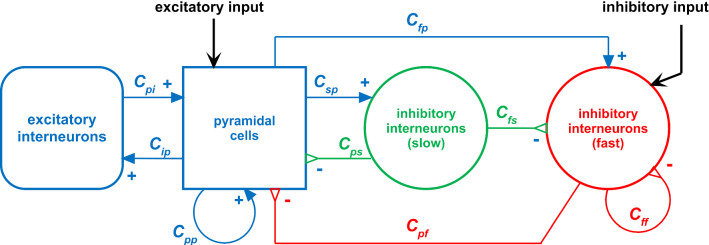
Scheme of the neural mass model simulating the dynamics of a single column. Blue continuous lines with arrows indicate glutamatergic excitatory synapses, red lines with open triangles indicate GABAergic faster inhibitory synapses, while green lines with open triangles indicate GABAergic slower inhibitory synapses. Symbols *C*_*ij*_ denote the synaptic contacts among the neural populations, where the first subscript and the second subscript designate the post-synaptic population and pre-synaptic population, respectively

The basic element of the model is a cortical column (Fig. 1): its output activity oscillates thanks to the local interaction among excitatory and inhibitory populations, arranged in feedback. As a fundamental point, we assume that the activity of a single cortical column represents an individual feature of an object (or item). With the term “object” in the following we will indicate any representation in memory consisting of multiple features, not necessarily a real object, but also a place, an event, a concept or any other mental construct. Briefly, an object is represented as a collection of features, each coded by the activity of the corresponding cortical column.

**Fig. 2:**
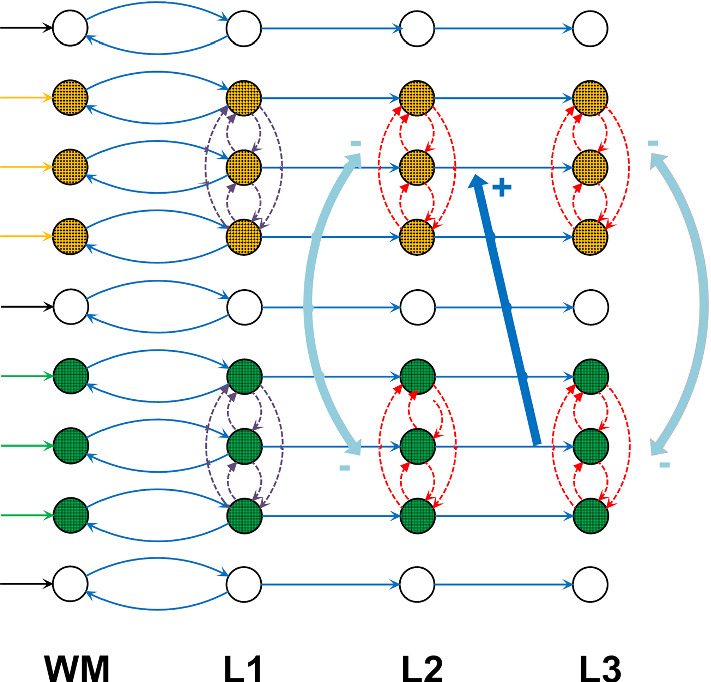
Schema of the different layers used in the present model, in which cortical columns representing different features are shown with an open circle. For the sake of simplicity, the features are arranged in a monodimensional chain in each layer. The simplified figure assumes two different objects, each composed of three features, denoted with different filling colors (orange and green). Continuous blue lines and dash-dotted violet lines represent long-range glutamatergic synapses of type *W*, connecting pyramidal to pyramidal neurons (hence excitatory); dash-dotted red lines represent synapses of type *K*, connecting pyramidal neurons to fast inhibitory interneurons in the same object (hence inhibitory via a bi-synaptic connection). Cyan lines represent fast synapses of type *A*, connecting pyramidal neurons to fast inhibitory interneurons in different objects (hence inhibitory via an ultrafast bi-synaptic connection). Note that, to make the plot simpler, we used large arrows to summarize a vector of synapses connecting the three columns in one object to three columns in another object (hence, the cyan fast inhibition line vectors connect three columns in one object to the three columns of another object within layer L2 and within layer L3; the blue excitatory line vector connects three columns of one object in layer L3 to three columns of the subsequent object in layer L2, assuming that the green object precedes the orange object in a stored sequence)

The overall network consists of four layers (see Fig. 2), arranged in a sequence (in the following, named layers WM, L1, L2 and L3). A hypothesis on where these layers may be located, and a partial justification of the connectivity patterns proposed, is reported below, with reference to the prefrontal cortex-hippocampus interactions, and further summarized in Table 1. A critical analysis is provided in the Discussion session.

**Table 1:** Mechanisms included in the model and their possible neurobiological counterparts

Layer	Functions modeled	Possible location	Necessary model components	Possible biological mechanisms/ pathways
WM	Maintainance of information in working memory Resetting of information	Medial prefrontal cortex + thalamic loop	External input Reentrant connections with L1 Resettable positive feedback	Bidirectional connections between the PFC and the perirhinal cortex or lateral entorhinal cortex (Eichenbaum 2017) Connectivity with the mediodorsal thalamus (Bolkan et al. 2017; Nir-Cohen et al. 2020)
L1	Memorization of objects Reconstruction of objects with lacking information Generation of the theta rhythm (sequence modality only) Help WM to maintain information in memory Transmission of the reconstructed objects to L2	Entorhinal cortex and CA3	Reentrant connections (not trained) with the WM Reentrant excitatory connections within an object, which are trained with the Hebb rule Output feedforward connections (not trained) to L2	Bidirectional connections between the PFC and the perirhinal cortex or lateral entorhinal cortex (Place et al. 2016; Eichenbaum 2017) Existence of Hebbian plastic excitatory synapses in the hippocampus (especially CA3) (Bliss and Collingridge 1993; Li et al. 1994) Existence of a feedforward connectivity from the entorhinal cortex to CA3 (Eichenbaum 2017)
L2	Generation of the gamma rhythm Segmentation of multiple objects with gamma temporal division Synchronization of the gamma and theta rhythms	CA3 + external loop (Papez’s circuit, septum)	Input (not trained) from L1 Local reentrant inhibitory connections within an object trained with the Hebb rule Local very fast inhibitory connections among different objects, trained with a anti-Hebbian mechanism Feedback mechanism which disinhibits neurons and synchronizes the theta rhythm Feedforward output (not trained) to L3 Input (trained with Hebbian mechanims) from L3	Existence of reentrant connections in CA3 with Hebbian plasticity (Bliss and Collingridge 1993; Li et al. 1994) Existence of gap junctions among pyramidal neurons in the hippocampus (Schmitz et al. 2001; Ixmatlahua et al. 2020) Demonstration of training mechanisms among gap junctions (Cachope et al. 2007; Turecek et al. 2014; Wang et al. 2015) Existence of a feedback mechanism between the hippocampus and external structures (Papez’s’s circuit, septum) causing a resonant theta rhythm (Vertes et al. 2001; Kocsis and Kaminski 2006; Salib et al. 2019; Dillingham et al. 2021) Schaffer collateral (Hongo et al. 2015; Kwon et al. 2018) Feedback synapses from CA3 to the entorhinal cortex (Craig and Commins 2005; Sandler et al. 2015; Eichenbaum 2017)
L3	Improving synchronization and desynchronization with the gamma rhythm Reconstruction of a temporal ordered sequence	CA1	Input (not trained) from L2 Local reentrant inhibitory connections within an object trained with the Hebb rule Local very fast inhibitory connections among different objects, trained with a anti-Hebbian mechanism Trained feedback synapses to L2, trained with Hebbian mechanism Output toward other brain structures	Schaffer collateral from CA3 to CA1 (Hongo et al. 2015; Kwon et al. 2018) Plasticity of synapses in CA1 (Tetteh et al. 2019) Existence of feedback connections from CA1 to the entorinhal cortex (and from there to CA3) (Craig and Commins 2005; Sandler et al. 2015; Eichenbaum 2017) Role of CA1 in the memorization of temporal aspects (Hoge and Kesner 2007; Mankin et al. 2012)

The same cortical columns (hence the same object) are replicated in each layer. We assume that each of the layer executes a computational step: to maintain the object in working memory (layer WM), to reconstruct lacking information (layer L1), to segment different objects in gamma band (layers L2 and L3) and, in case of a sequential order among objects, to reproduce the correct order of objects with a gamma code nested in the theta rhythm (layers L2 and L3). Fixed feedforward synapses (i.e., not subject to training) exist from a cortical column in each layer to the corresponding cortical column in the subsequent layer (as shown in Fig. 2), with the aim to propagate information downstream. Furthermore, fixed synapses exist from a column in L1 and the corresponding column in WM, to help working memory maintenance and to send reconstructed information back. Feedback synapses (either within the same layers L1, L2 and L3 or from a downstream layer L3 to the upstream layer L2) are subject to training, and are used to store and recover objects, thus realizing auto-associative and hetero-associative memories.

We are aware that the use of fixed feedforward synapses may appear as a limitation of the model, in contrast with what is done in modern neural networks. However, this choice has been adopted for the sake of parsimony and to simplify the analysis of results. First, we assumed that each element in a layer describes a specific feature of the object, and we use the same features in all layers. Hence, our idea is that each feature transmits information of its status (active or silent) to the subsequent layer to instantiate a further processing without modifying the features. Conversely, in several biologically inspired networks, training of feedforward synapses allows the extraction of more complex features in subsequent layers, starting from simpler features in previous layers; in other words, a more basic object representation in initial layers is translated into a more abstract representation downstream (Aggarwal 2018). Of course, our choice is parsimonious, thus reducing the number of parameters subject to training. For a similar reason, the synapses from L1 to WM are fixed, since they only have the function to maintain a closed loop self-sustained memory between WM and L1, without any further processing step.

Of course, a network in which feedforward synapses can also be trained may exhibit more complex patterns of activity and more powerful behavior; this can be the subject of future model extensions. By the way, we are not aware whether this abstraction processing schema (from simpler to more complex features), which is typical of the visual temporal pathway, can also occur in the hippocampus processing stream.

### The model of a single cortical column

**Fig. 3:**
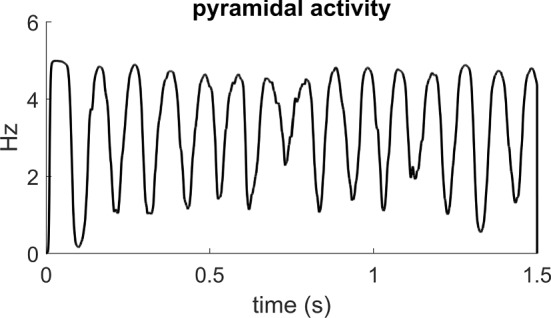
Example of the activity (spike density) in the population of pyramidal neurons when a column is not connected with any other column, and is stimulated with a white noise input. The oscillations belong to the alpha range (about 10 Hz)

A column consists of the feedback arrangement of four neural populations, according the schema depicted in Fig. 1. These are pyramidal neurons, excitatory interneurons, GABA-ergic inhibitory interneurons with slow synaptic dynamics, and GABA-ergic inhibitory interneurons with fast synaptic dynamics. A more detailed description of the column is provided in previous papers of the authors (Ursino et al. 2010; Cona et al. 2011, 2012). It is worth noting that we adopted the same parameters for each cortical column in each layer, i.e., differences in rhythms between one layer and another originate from the presence of feedback synapses among the columns, produced by the training procedure. Indeed, with the present parameter values a single column, if isolated from the others, and stimulated by a constant external input, produces an intrinsic oscillation in the alpha band (see Fig. 3).

Just in the case of layer WM, which implements working memory (possibly located in a structure of the frontal lobe) we added a further auto-excitation loop for pyramidal neurons (i.e., a group of pyramidal neurons can auto-excite themselves via parameter *C*_*pp*_ in Fig. 1). This loop is necessary to keep information in memory during the maintenance period of a working memory task, and is reset to zero whenever a new input updates the memory content. From a neurophysiological perspective, the excitatory closed loop among pyramidal neurons included in WM (and not used in the other layers) can be ascribed either to reverberations within local cortical circuits, or to long-range circuits (Guo et al. 2017; Zylberberg and Strowbridge 2017).

Finally, it is worth noting that each cortical column can receive two different inputs, the first directed toward pyramidal neurons, with an excitatory function, and the second toward fast inhibitory interneurons, with an inhibitory role. Both inputs can be affected by noise. The output of each column is the spike density of pyramidal neurons (ranging between 0 and 5, as in previous models, see Jansen and Rit (1995)).

### The individual layers

We used a four-layer structure in our model, arranged as in Fig. 2, but with two possible alternative uses: the first, named “**sequence ordering working memory**”, aims to recover a previously learned sequence of items in an assigned temporal order, by exploiting a theta-gamma coupling mechanism. The second, named “**semantic working memory**”, aims to maintain several objects simultaneously in memory, by desynchronizing them in the gamma range. Since some works in the literature (see Discussion) suggest that the theta rhythm is important especially in sequence ordering, in the second model use we do not generate the theta rhythm. In other words, we implement two different networks in this work, to solve two distinct problems. However, the two networks exhibit the same topology and share most internal parameters, and differ only for what concerns a few synapses. It is possible that these networks represent processing in different parts of the hippocampus. Indeed, some experimental evidences suggest that the hippocampus is functionally differentiated between the dorsal (posterior) and ventral (anterior) areas (Moser and Moser 1998) and that distinct hippocampal-cortical connections are one mechanism by which the hippocampus can represent different kinds of knowledge (Frank et al. 2019).

The two kinds of networks differ only for what concerns two aspects: first, the synapses between WM and L1 are three-times stronger in the “semantic” modality than in the “sequence ordering” modality: in this way, the pyramidal neurons in L1 are excited to saturation and a theta rhythm is not produced. This difference agrees with the presence of two parallel streams in the hippocampus (one named “what” stream and other “where” stream) as described in Preston and Eichenbaum (2013). In particular, the latter authors assert that the medial prefrontal cortex, in the “what” streams, is positioned to influence the retrieval of specific object representations via its particularly *strong connections* to perirhinal and lateral entorhinal cortex, whereas connections are smaller in the “where” stream (see Fig. 1 in their work). Second, feedback synapses from L3 to L2 are trained only in the “sequence ordering” modality, to produce a sequence of items from an initial cue, but are set at zero in the “semantic” modality. All other aspects of the model are equal in the two modes.

Each layer consists of an array of *L*x*M* identical cortical columns (hence, we can have *L*x*M* different features) arranged in a regular lattice. As said before, the only difference among the columns is the presence of an auto-excitatory loop in pyramidal neurons of layer WM. In the present simulations we used *L* = *M* = 20 (hence, we have a total of 400 distinct features).

In the following, three different kind of synapses will be used, to connect columns within a layer or among different layers:

i. glutamatergic excitatory synapses (named *W*) from pyramidal to pyramidal neurons;
ii. glutamatergic excitatory synapses (named *K*) from a pre-synaptic pyramidal population to a post-synaptic fast-inhibitory population. Since these synapses target inhibitory interneurons, their overall effect is inhibitory on the pyramidal population of the post-synaptic column;
iii. synapses (named *A*) from a pre-synaptic pyramidal population to a post-synaptic fast-inhibitory population, but with much faster dynamics. The overall effect is still inhibitory, but occurs almost immediately (much earlier than the effects in points i and ii).

Values of the time constants for these three synaptic mechanisms are about 7.7 ms, 6.8 ms and less than 1 ms, respectively.

The different speeds of the synaptic mechanisms in points i and ii, compared with point iii, can be ascribed to the presence of differences in AMPA receptors in the postsynaptic membrane. Another stronger possibility is that faster dynamics are mediated by gap junctions.

#### Layer WM

Even though the present network does not aspire to reproduce individual regions of the brain−rather, it provides a general framework for the role of brain rhythms in memory—it is thinkable that the first layer represents a region in the prefrontal cortex, which starts implementing working memory. Indeed, several results in the literature suggest that memory tasks are characterized by synchronized neural activity between hippocampus and ventromedial prefrontal cortex (vmPFC) (see Eichenbaum (2017)), that the vmPFC activity leads that of the hippocampus (Barry et al. 2019) and that both regions are critical for simulation of future episodes, hence to memorize temporal order (Campbell et al. 2018).

Each cortical column receives an external excitatory input: the latter may be zero (i.e., there is just noise) signifying that the particular feature is absent, or may have a high value, signifying that the feature is present. The aim of layer WM is to maintain one or more objects (previously learned) in memory, even after the corresponding inputs have been removed. To this end, besides receiving auto excitation via synapses *C*^*pp*^ (see Fig. 1), pyramidal neurons in layer WM also receive an excitatory feedback (not trained) from the corresponding columns in layer L1 (see Fig. 2). This has two functions: to reinforce the content maintenance in working memory, with an additional excitatory loop, and to allow the restoration of an entire object, by recovering its lacking elements. In fact, reconstruction is realized in layer L1 (which works as an auto-associative memory and may be located in the hippocampus, see below) and the resulting information is transmitted back to WM. Hence, WM maintains in memory both the information received from the external inputs, and the information reconstructed in L1, even when the external output is set to zero. The presence of bilateral connections between the PFC and the hippocampus, playing a role in oscillatory synchronization and learning is well documented (see Eichenbaum (2017), pp. 550–552).

However, as soon as a new external input is presented to the WM layer, the previous content must be reset. The idea that working memory must receive a reset signal whenever its content has to be adjourned found wide support in the recent literature (Balaban and Luria 2017; Balaban et al. 2018). In particular, working memory needs to protect its current content from interference, and simultaneously must be able to update this information rapidly. As described in Nir-Cohen et al. (2020) a possibility is that these opposing requirements are met via a Basal Ganglia-Thalamus positive feedback loop.

In our model, the mechanism proposed by Nir-Cohen et al. (2020) for selective updating of PFC working memory representations is implemented through the connection *C*_*pp*_, assuming that these schematize the Thalamic-Basal ganglia mechanism. Indeed, action channels in the Basal Ganglia are generally segregated, which justifies the use of several parallel reentrant loops. Hence, as soon as a new external input is presented to the WM layer, the auto-feedback synapses of the pyramidal neurons, *C*_*pp*_, are set to zero to allow a refresh of the working memory content. Of course, this is a simplification. A more complete Thalamic-BG circuit, similar to that described in Baston and Ursino (2015) can be added in future model developments.

The output of layer WM is directed toward the corresponding cortical column (i.e., the column in the same position) in layer L1, with constant synapses. It is worth noting that no synapse is trained within layer WM, i.e., this layer only aims to maintain information for a short period, and does not implement any form of long-term memory. We are aware that synapses within the PFC are plastic, and their plasticity contributes to the role of the PFC in planning and control. A top-down control of the PFC over the hippocampus for organization of memory and planning is well documented (Eichenbaum 2017). However, since these functions are not included in our simplified model, we do not incorporate plasticity within the PFC. This may be added in future works, if trials involving a top-down control are simulated.

#### Layer L1

Layer L1 works as an auto-associative memory, to restore a complete object in memory starting from an incomplete cue. Moreover, after training this layer also produces the theta rhythm.

Each cortical column in L1 receives its excitatory input from the corresponding column in layer WM. During a training phase (see below), when individual objects are presented for long-term storage, further excitatory synapses of type *W* are created via a Hebbian mechanism, linking columns simultaneously active in the same object. Like in classical auto-associative networks, the synapse matrix so-created is symmetrical (Hopfield 1984). The presence of these synapses causes a prolonged excitation in the pyramidal population, which is then transmitted to the population of slow inhibitory interneurons; the latter, in turn, transiently inhibit the pyramidal population inducing a slow theta rhythm. The presence/absence of such a rhythm can be controlled acting on the strength of the synapses (both lateral synapses, and those arriving from WM). However, in order to allow the generation of a similar rhythm independently of the object size (i.e., we wish that objects containing a different number of features exhibit the same rhythm) we assumed that the sum of the synapses entering into a given cortical column is constant: hence, during training the sum of synapses is normalized to a given saturation level. This is a physiological mechanism, often adopted in previous neural network models (Ursino et al. 2015). Thanks to this normalization, objects with different dimensions receive a similar input and oscillate at the same frequency.

The output of pyramidal neurons from L1 is sent back to WM, to instantiate working memory, and transmitted to layer L2 (as described below) with fixed (i.e., not trained) synapses.

Since in our model the layer L1 is connected with the PFC and realizes an auto associative memory, it probably involves the entorhinal and/or perirhinhal cortex and the CA3 region, where feedback synapses are known. Actually, the presence of bidirectional connections between the PFC and the lateral entorhinal cortex/the perirhinal cortex are well documented (see Eichenbaum 2017). Moreover, several results support the idea that these connections bidirectionally exchange theta information (Siapas et al. 2005; Place et al. 2016) as in the present model.

#### Layer L2

This layer, together with the downstream layer L3, realizes a desynchronization of different objects in the gamma band and, in case of a sequence of objects, nests the sequence within the on phase of the theta rhythm. Each column in this layer receives a fixed excitatory synapse from the corresponding unit in L1. Moreover, as explained below, in case of a temporal sequence, L2 also receives feedback excitatory synapses from columns in L3.

We think this layer may be located in CA3. In fact, as reported in Zemankovics et al. (2013) gamma frequency oscillations can be generated intrinsically in the CA3 region of the hippocampus from where they can propagate to the CA1 area. Montgomery and Buzsáki (2007) propose that gamma oscillations may serve as a physiological mechanism by which CA3 output can coordinate CA1 activity to support retrieval of hippocampus-dependent memories. Conversely, as described in Eichenbaum (2017) a feedback from CA3 to the entorhinal/perirhinal cortex does not seem to play a relevant role, thus justifying the absence of a feedback from L2 to L1 in our model.

Clearly, in order to realize a correct segmentation of different objects in the gamma band, two fundamental conditions must be met: (i) synchronization of the activity for all features belonging to the same object: i.e., all units in the same object must oscillate with approximatively the same phase; (ii) desynchronization of activity of features in different objects: i.e., units belonging to different objects must oscillate out of phase.

Previous works (Terman and Wang 1995; Campbell and Wang 1996; Ursino et al. 2009) demonstrated that, in order to produce a robust synchronized activity, reciprocal inhibition between units is more appropriate than a reciprocal excitation. Hence, we assumed that, during training, synapses of the type *K (*thus *producing inhibition),* are reinforced in layer L2 via a Hebbian mechanism, linking cortical columns in the same object (see section below). It is worth noting that, thanks to the presence of this reciprocal inhibition, units in this layer start oscillating with a frequency in the gamma range (about 30 Hz) and in a highly synchronized way. The mechanism is as follows: a group of units in a given object is initially excited from the input coming from layer L1 to layer L2; these units inhibit reciprocally, causing a rapid reduction in their excitation; once the excitation has faded away, also the reciprocal inhibition falls down, and the cycle starts again. Since the dynamics of the fast inhibitory interneurons is in the same range as the gamma rhythm, this mechanism causes an oscillation in this frequency band.

Several papers support the idea that GABAergic interneurons play a relevant role in maintaining network oscillations, especially in controlling the precision of spike timing (Lasztóczi and Klausberger 2014; Geng et al. 2018).

However, using this mechanism only, multiple objects simultaneously in memory would be superimposed, i.e., they would oscillate with the gamma frequency but without achieving a correct segmentation. In order to segment objects, we used further synapses of type *A* (i.e., causing inhibition, but with much faster dynamics). These are trained with an anti-Hebbian mechanism. This means that these synapses are reinforced when the presynaptic population (pyramidal neurons) is active and the post-synaptic population (fast inhibitory interneurons) is silent. In this way, a very fast inhibition is created from units in one object to all units in different objects. Thanks to this rapid mechanism (that we assumed almost instantaneous) as soon as an object emerges in layer L2, all other objects are inhibited, thus realizing a good segmentation.

Various data in the literature suggest the presence of distinct inhibitory mechanisms working in the hippocampus. Although some of these mechanisms have been analyzed especially with reference to CA1 cells, in our model they work in a similar fashion both in L2 and L3 layers, hence we discuss them here. In particular, results by Balakrishnan and Pearce (2015) indicate that multiple distinct local circuits generate γ-oscillations in the CA1 region of the hippocampus. Jang et al. (2015) analyzed the role of diverse variety of hippocampal interneurons, which provide either feedforward or feedback inhibition to CA1 pyramidal cell and observed that they differentially modulate the gain, the spike precision, the neural code transformation and the information capacity. Results by Butler et al. (2018) indicate that a fast excitatory-inhibitory feedback loop underlies the generation of gamma oscillations in the hippocampal regions. Data by Klausberger and Somogyi (2008) suggest that distinct GABAergic cell types interact with glutamatergic pyramidal cell inputs and support synaptic temporal dynamics, network oscillations and selection of cell assemblies.

Nevertheless, although the results mentioned above support the presence of multiple circuits, exploiting fast interneurons to produce gamma rhythms, the presence of type A synapses remain one of the most hypothetical aspects of the present model. This mechanism is essential to desynchronize the gamma oscillations in different objects and so represent a strong prediction for future studies. A strong candidate can be the presence of gap-junctions. Various authors recently demonstrated the presence of an ultra-fast electrical communication mechanism in the hippocampus, and related it with network oscillations (Schmitz et al. 2001; Ixmatlahua et al. 2020).

The synapses *K* and *A* described above are also normalized after training, so that the sum of synapses entering into a column is constant.

Finally, results in the literature (Colgin et al. 2009; Lisman and Jensen 2013) suggest that, during the recovery of a list of objects from memory, a gamma-sequence is nested within the on phase of the present theta period, then breaks down during the off phase, to start again at the beginning of the subsequent theta period (see also the phase precession phenomenon (O’Keefe and Recce 1993; Skaggs et al. 1996; Tsodyks et al. 1996)). To realize this theta-gamma coupling, we need another mechanism. To this end, we assumed that all units in layer L2 are normally silenced: this is achieved with a strong external input sent to fast inhibitory interneurons in all columns. This inhibition is then removed, with very fast dynamics, as soon as the global activity coming from layer L1 overcomes a given threshold, a condition occurring in the sequence order modality with the theta frequency. As a consequence, neurons in layer L2 are active only during the ON phase of the L1 theta rhythms.

The presence of an external mechanism operating on theta finds several support in the literature. In particular, there are at least two external circuits active on CA3 which may serve this role. The first is the so-called Papez’s circuit, which involves the hippocampal formation, mammillary bodies, anterior thalamus, cingulate cortex, parahippocampal gyrus, hippocampal formation. It has been shown that cells of mammillary body fire in synchro with the theta rhythm of the hippocampus (Kocsis and Vertes 1994; Bland et al. 1995; Kirk et al. 1996) and that this rhythmical activity is dependent upon the action of the hippocampus on the mammillary bodies (Kocsis and Vertes 1994; Bland et al. 1995). It has been hypothesized that a theta-rhythmic signal may resonate throughout Papez's circuit, possibly involved in the control of mnemonic functions of the circuit (Vertes et al. 2001). Several data suggest that lesions of the mammillary bodies, mammillo-thalamic tract and anterior thalamic nuclei all produce severe impairments in temporal and contextual memory (Dillingham et al. 2021) thus supporting the idea that mammillary bodies are important for coordinating hippocampo-cortical activity. A second alternative circuit may involve the medial septum: Salib et al. (2019) proposed a mechanism involving septal low-rhythmic GABAergic firing neurons which innervate interneurons mostly in the dentate gyrus (DG) and CA3. The preferred firing phase of these septal neurons during theta oscillations matched the highest firing probability phase of principal cells in the DG and CA3, inducing a transient disinhibition of principal cells. It is worth-noting that this mechanisms strongly resembles the mechanism proposed in our model.

#### Layer L3

Briefly, all columns in layer L3 receive an excitatory input from the corresponding column in layer L2 (note that this input is already partly segmented and oscillates in the gamma + theta range). Lateral synapses among columns within layer L3 are trained with exactly the same mechanisms used to train layer L2; i.e., we reinforce synapses *K* among features within a given object via Hebbian mechanisms, and reinforce fast synapses *A* among features of different objects via anti-Hebbian mechanisms. This interaction in layer L3 strongly improves the previous segmentation (see results).

The presence of feedforward connections from CA3 to CA1, called Schaffer collateral, is well documented, and constitutes an important route for the information flow in the hippocampus. Hongo et al. (2015) observed that CA3 axons innervate CA1 neurons in a highly topographical fashion. Furthermore, while synaptic plasticity has been well documented in CA3 cells for many decades, several recent results suggest that synapse long-term potentiation occurs in CA1 too (Tetteh et al. 2019).

However, layer L3 is not only useful to improve segmentation, but it also plays an essential role to store a sequence of items in the correct order. To this end, when a list of objects must be memorized with a given temporal order (that is, only in the network used for sequence ordering), we create hetero-associative excitatory synapses (of the type *W*) from L3 back to L2. These link all features in L3 coding for the previous object (for instance the (k-1)th object in a list), to the features in L2 coding for the subsequent object (i.e., the kth object of the same list). In this way, after training, the activity of one object in layer L3 spontaneously evokes activity of the subsequent object in layer L2. The latter, in turn, is feedforwarded to layer L3, to continue scanning the list. These steps continue again and again, until the overall sequence of objects is terminated and/or the on phase of the theta rhythm is over.

Several results support the presence of feedback information from CA1 to CA3 (whereas a similar feedback is lacking or less important from CA3 to the entorhinal cortex, at least to our present knowledge). In particular, anatomical connection from CA1 to CA3 exist via the Entorhinal Cortex (EC) and through backprojecting interneurons (Eichenbaum 2017). Sandler et al. (2015) applied Granger causality to spike activity recorded in the CA3 and CA1 areas of the rodent hippocampus, and found a causal relationship not only from CA3 to CA1 but also from CA1 to CA3. Furthermore, it has been shown that the CA1 → EC pathway is able to undergo long-term potentiation, suggesting a role for this pathway in learning and memory (Craig and Commins 2005).

### Training the network

As specified above: (i) lateral excitatory synapses of type *W* are created among cortical columns within the same objects in layer L1, via Hebbian potentiation, to allow reconstruction of lacking information; (ii) lateral inhibitory synapses of type *K* are created among units within the same object in layers L2 and L3, via Hebbian potentiation, to allow synchronization in the gamma range; (iii) faster lateral inhibitory synapses of type *A* are created among columns in different objects in layers L2 and L3, via anti-Hebbian potentiation, to allow desynchronization; (iv) in case of a sequence of objects, excitatory synapses of type *W* are created from units in one object in layer L3 to units of the subsequent object in layer L2, via Hebbian potentiation, to allow the reconstruction of a sequence starting from the first item.

Training consists in the presentation of the objects to the layers, one after the other, as specified below, with excitatory inputs so strong that all cortical units reach a saturation level (i.e., the columns do not oscillate during training). The Hebb and anti-Hebb rules are then applied after the units have reached a steady state level.

To realize the connections described above, we implemented two different training steps, the first valid for both networks, to memorize individual objects, the second for the sequence ordering network only, to memorize those objects in an assigned sequence.

(i) During the first step we provided each object separately to layers L1, L2 and L3; this is obtained by stimulating the corresponding pyramidal neurons in layer L1, and both the pyramidal neurons and the fast inhibitory interneurons in L2 and L3. Synapses of type *W* are then created in L1 using the Hebb rule (see Eqs. (11) and (12) in the Appendix), synapses of type *K* are created in L2 and L3 using the Hebb rule (see Eqs. (13) and (14) in the Appendix), whereas synapses of type *A* were formed with an anti-Hebbian rule (see Eqs. (15) and (16) in the Appendix). This procedure required 2000 epochs.

After this training, the network can be used in a modality named “**semantic working memory**”. The objective here is to maintain different objects simultaneously in memory, each segmented in the gamma band.

(ii) Only if a sequence of *N* objects must be memorized in an assigned temporal order (i.e. one needs to store and recover an ordered list of items) a further training step was performed, involving layers L2 and L3 together. This is coherent, thinking to a task in which first we memorize some items, and then memorize a particular sequence for the same items. In particular, naming the objects in the list as: *Obj_1, Obj_2*, …, *Obj_N* during each training step the following inputs are provided:

layer2: *Obj_k*; layer3: *Obj_(k-1)* (with *k* = 2, 3, …*N*).

To this end, an input was provided to the corresponding pyramidal neurons in the two layers. By applying the Hebb rule (see Eqs. (17) in the Appendix) hetero-associative synapses are created from *Obj_(k-1)* in layer L3 to *Obj_k* in layer L2. This procedure required 1000 epochs.

After the step ii, the network can be used in a modality named “**sequence-ordering working memory**”. The objective here is to recover an entire sequence, starting from a first incomplete item, and to nest this sequence within the on phase of each theta cycle.

To recapitulate all previous considerations, a list of the main functions introduced in the model, the necessary model components for these functions, and possible neurobiological evidences in the literature, with reference to the PFC-hippocampus interactions, is presented in Table 1.

## Results

**Fig. 4:**
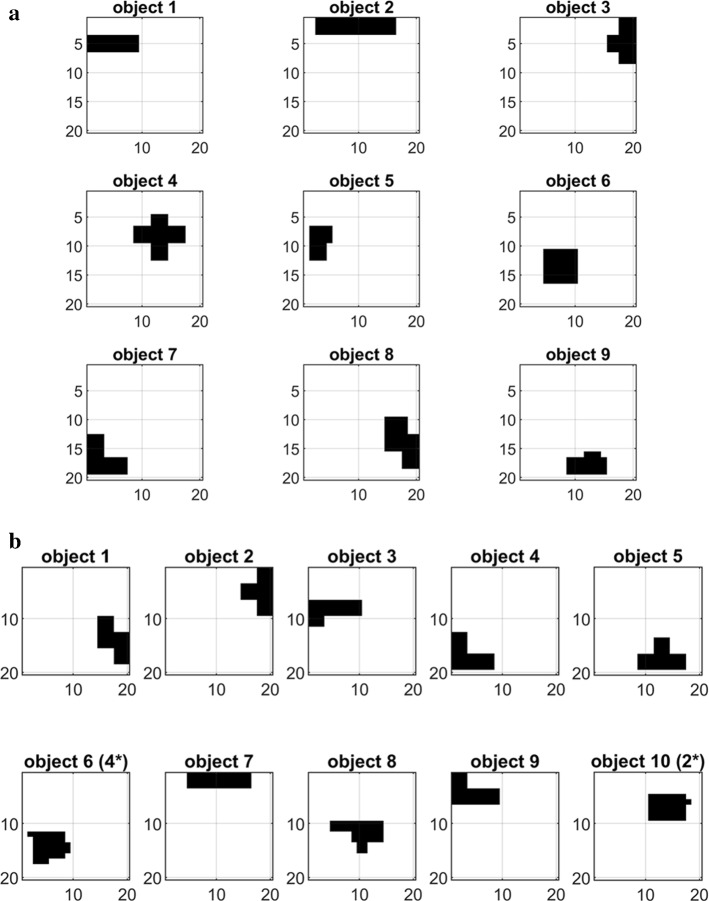
Combinations of objects used during the present simulations. The combinations presented in panel *a* includes nine different objects, with different dimensions but orthogonal (i.e., without any common feature). The configuration in panel *b* presents ten different objects, with the same dimensions but overlapping features. In particular *object 2* has 20% of feature overlapping with *object 10*, and *object 4* has 20% of features overlapping with *object 6*

Two different collections of objects have been used to train the network and to test its behavior. The first, shown in the upper panel of Fig. 4 and named *collection 1*, is composed of nine objects (or patterns) with different dimensions: each object is orthogonal to the others, i.e., objects have no common pixel. The second collection, represented in the bottom panel and named *collection 2*, is composed of ten objects with identical dimensions but now with up to 20% common pixels. In particular, the object 6 is partially superimposed to object 4, and object 10 to object 2. All results are repeatable with both collections of objects in the sequence order modality, whereas in the semantics modality only orthogonal objects were used. In the following, results in the figures will be shown with reference to collection 1; only in case of the last section (“dreaming”) results are presented with reference to the second collection, to exploit the partial superimposition among objects. It is worth noting that we used connected objects just for simplicity, but this assumption is not essential. Features of an object can be at any positions in the network, without any change in the model behavior.

Finally, as illustrated above, all objects have been trained in layers L1, L2 and L3. Moreover, in the configuration “sequence ordering” the model was further trained to memorize an ordered sequence: in the first configuration all nine objects are included in a single sequence, with order “1–2–3–4–5–6–7–8–9”; in the second configuration, two separate sequences, with a partial overlapping, were trained, i.e., the sequence “1–2–3–4–5”, and the sequence “6–7–8–9–10”.

Finally, although objects are presented in Fig. 4 in a two-dimensional lattice, in order to simplify the implementation all matrices were converted into one-dimensional vectors. For this reason, in the Appendix each cortical column is described with one subscript, while all synapses are described with two subscripts.

### Working memory

**Fig. 5:**
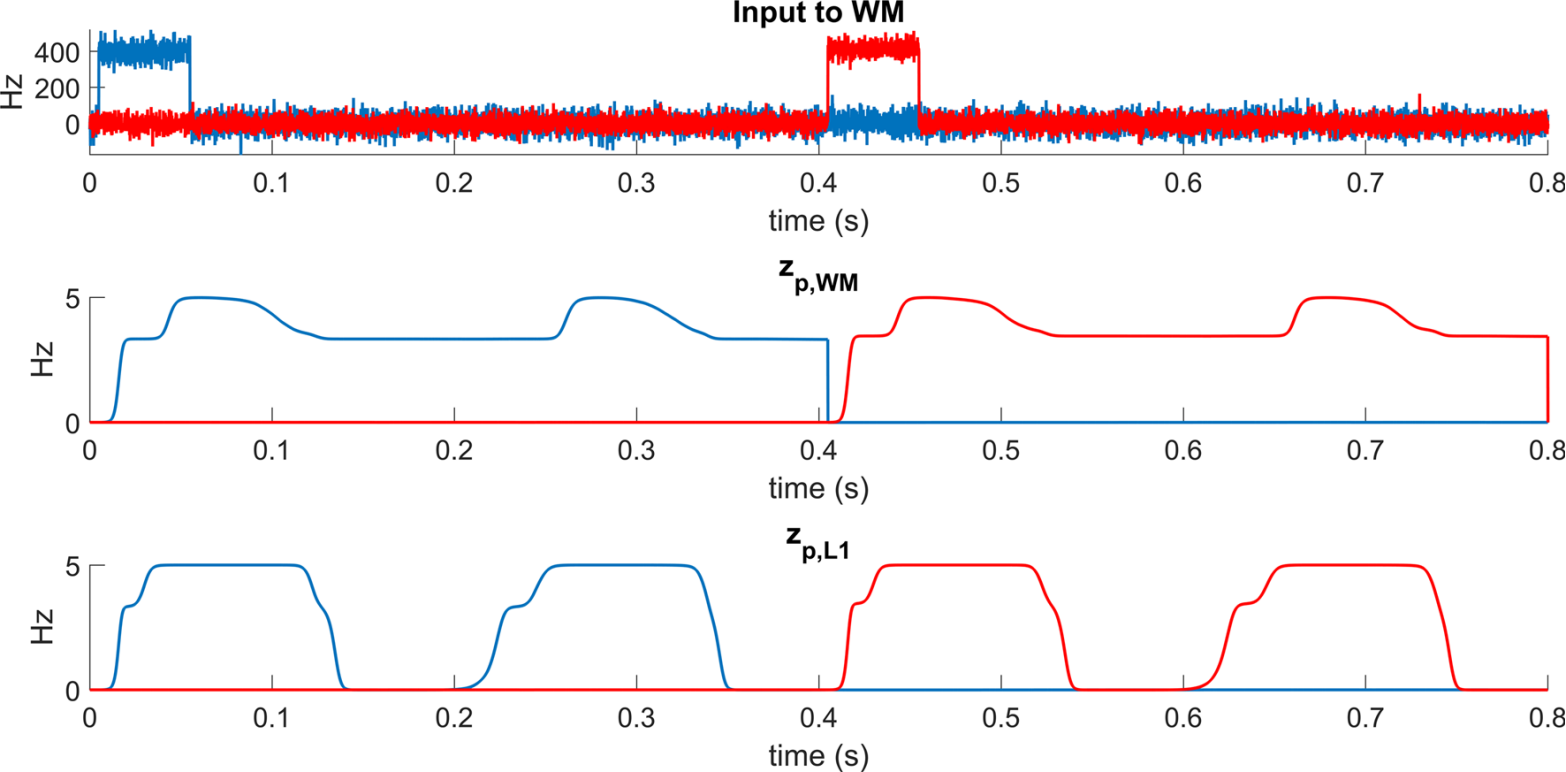
An example of the behavior in the WM and L1 layers in feedback. The simulation presents the effect of two separate inputs, provided to the WM layer per 50 ms, between the instants 0.005 and 0.055 s (70% of features are excited in object 1 blue line), and between 0.405 and 0.455 s (70% of features excited in object 2, red line). The variables z_p_ in layers WM and L1 represent the average spike density of all columns in that object (a value 5 means that 100% of features are excited in the object, a value 3.5 means 70% of features excited). Simulation shows that the activity in layer WM is maintained also when its input is zero, and is reset at the presentation of a new object. The activity in L1 oscillates with the theta rhythm, reconstructing all lacking features in the object during the on phase of the rhythm, and sending this information back to WM

In order to illustrate how an object can be maintained in working memory, even after removal of the corresponding input, Fig. 5 shows the input to the network, and the activity of pyramidal neurons in layers WM and L1, after the separate presentation of two objects (in this particular example object 1 is presented per 50 ms between the instants 0.005 and 0.055 s, and the object 2 is presented between 0.405 and 0.455 s, upper panel). Both objects are incomplete, i.e., they lack 30% of their pixels. The second and third panels of the figure represent the *average activity* of all pyramidal neurons coding for the object 1 (blue line) and for the object 2 (red line). Since the maximal activity in the model saturates at a value 2*e*_0_ = 5 (see the Appendix), an average activity as great as 5 means that all neurons are simultaneously active in a given object, while an average activity as large as 4 means that 80% of neurons in that object are excited, and a value close to zero means that all neurons are silent. As it is clear form Fig. 5, 70% of neurons in the object 1 are initially excited in response to the input and this information is transmitted to layer L1 through the feedforward synapses. Thanks to the presence of auto-associative synapses in L1, trained with the Hebb rule, layer L1 is able to recover all lacking information. The activity in layer L1 oscillates with a theta rhythm (approximately 5 cycles/s) and during each cycle the overall object is reconstructed in this layer. It is worth noting that information is permanently maintained in memory in layer WM, thanks to the presence of auto-excitation between pyramidal neurons. At the presentation of the second object (instant 0.405 s) this auto excitation is reset to zero, thus allowing a refresh of the memory, so that a second object is reconstructed in L1 and maintained in memory in WM.

### Reconstruction of a sequence of events and phase precession

**Fig. 6:**
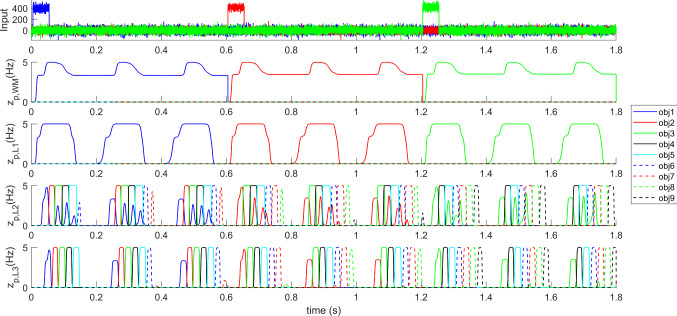
Recovery of a list of objects and phase precession in the modality “sequence ordering memory”. During the simulation a brief 50 ms excitatory input is given to 70% of features of *object 1* in WM layer between the instants 0.005 and 0.055 s. As a consequence, the network in L3 reconstructs the initial sequence of objects “1–2–3–4-5–6” nested within the theta cycle, and maintains this sequence even after the cessation of the input stimulus, until a new stimulus is given. Subsequently, a brief presentation of 70% of features of *object 2* is given between the instants 0.605 and 0.655 s, causing the appearance of the sequence “2–3–4–5-6–7-8” nested within the theta cycle. Finally, a brief presentation of 70% of features of *object 3* between the instants 1.205 and 1.255 s recovers the sequence “3–4–5–6–7–8–9–10”. Note the occurrence of phase precession when the input shifts from 1 to 3

Figure 6 shows how the model can work in the “sequence ordering” modality, i.e. after a sequence was learned during the step iii of the training procedure. After a brief 50 ms presentation of object1 (between the instants 0.005 and 0.055 s), the network is able to reconstruct the initial portion of the sequence, from object 1 to object 6: each object is coded by an individual period in the gamma cycle (note the different colors, representing activity within different objects) nested within the lower-frequency theta cycle. The sequence is then maintained in memory, and repeated periodically, until a new input is given to the network to refresh the WM content. In particular, the brief presentation of the second object (between the instants 0.605 and 0.655 s) allows the reconstruction of the sequence from object 2 to object 8, and the quick presentation of the object 3 (between the instants 1.205 and 1.255 s) produces the reconstruction of a sequence from object 3 to object 9. The well-known phenomenon of phase precession (similar to that observed in the rat’s spatial cells during movement) is evident looking at the phase of the different objects within the theta cycle.

From the figure the role of the different layers is evident: just a single object is reconstructed in layer L1 (by recovering all lacking information), where the theta rhythm is formed. A noisy gamma sequence is first produced in layer L2, and significantly polished in L3, where the reconstruction of all subsequent objects in the sequence turns out almost perfect.

### Desynchronization of several simultaneous objects

**Fig. 7:**
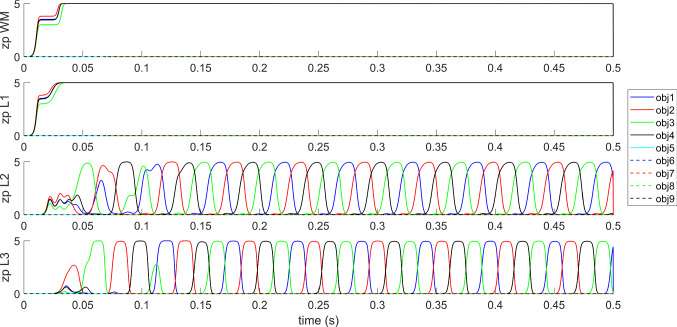
Simulation in the modality “semantic memory”. Note that, in this modality, thanks to the use of greater synapses between WM and L1, the activity in L1 does not oscillate with a theta rhythm. An excitatory input is given to 70% of pixels in four different objects (*object 1*, *object 2*, *object 3* and *object 4*) in WM per 50 ms. The network can maintain all objects in memory; the activity in L3 exhibits a desynchronization of all features in the four objects, assigning a constant temporal sequence (in this particular case the sequence is object1—object 2—object 4—object 3, but it can change from one simulation to the another due to noise realization). It is worth noting that, in this modality, this sequence was not learned by the network, i.e. we do not have feedback synapses from L3 to L2

The previous simulation illustrated the functioning of the model in the “sequence ordering” modality, when a sequence of events can be reconstructed in the correct order, starting from the first element of the list (and, moreover, the first element can shift in time). Figure 7 shows the model working in the “sematic memory” modality (i.e., step ii was not performed in the training procedure, and synapses between L1 and WM are stronger). In this particular example, four objects are simultaneously given as an input, and the model is able to desynchronize their activity in the gamma range, so that objects appear separately (that is, in time division) in layer L3.

An aspect deserves attention. In this modality no sequence order was previously memorized (i.e., we did not train the feedback synapses from L3 to L2), and so we were not requiring any particular order to be reproduced. Nevertheless, the model “devises” a particular order (in the specific example, the order is “object 1—object 2—object 4—object 3”) and repeats this order constantly, after a brief transient. This order can change as a consequence of the particular noise realization.

This kind of behavior (i.e., a correct desynchronization with a fixed although arbitrary order) can be observed, with the assigned synapse values, when either two, three or four objects are simultaneously given as input. We also tried to desynchronize a greater number of simultaneous objects with the same values of synapses, but, if the number of objects was greater than four, not all objects could be desynchronized correctly.

**Fig. 8:**
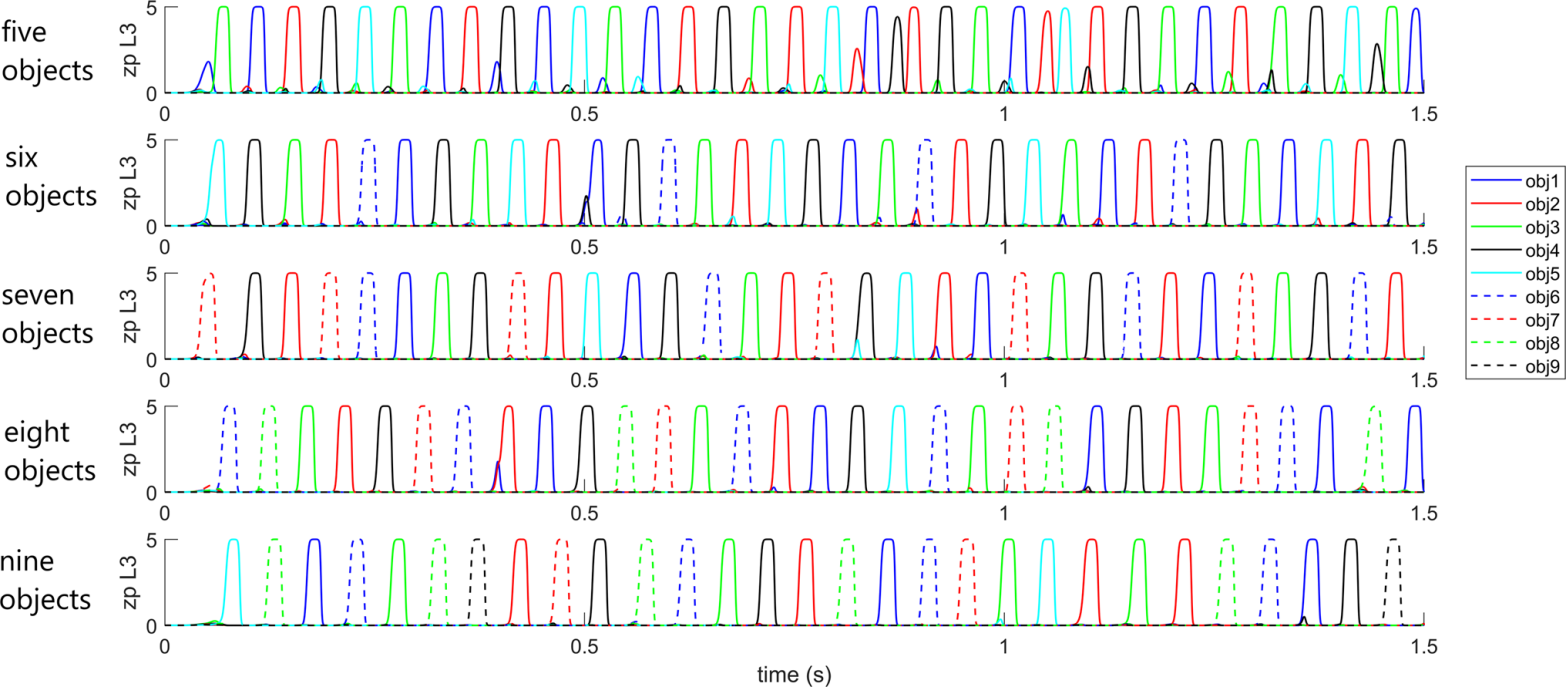
Simulation in the modality “semantic memory”. In this figure, only the average spike density of the different objects in layer L3 is presented for brevity. The five panels represent model response when 5, 6, 7, 8 or 9 objects are simultaneously used as input in WM per 50 ms. The network maintains all objects in memory and desynchronize them, but without maintaining any constant order (the frequency and positions of the objects can change with time). Note that, in these simulations, the strength of the fast synapses ${A}_{ij}^{{L}_{2},{L}_{2}}$ and ${A}_{ij}^{{L}_{3},{L}_{3}}$ has been increased by a factor as high as 1.7 compared with the value used in Fig. 7, which justifies the smaller frequency as a consequence of an increased competition among objects

However, we observed that the capacity to desynchronize objects improves significantly if the strength of the synapses *A* in layers L2 and L3 (i.e., synapses ${A}^{{L}_{2},{L}_{2}}$ and ${A}^{{L}_{3},{L}_{3}}$ see the Appendix) is increased. In particular, up to nine objects could be desynchronized rather often by multiplying the previous synapse values by a factor in the range 1.25–1.7 (*A*_*max*_ = 0.15–0.20). Some examples, concerning the desynchronization of 5, 6, 7, 8 and 9 objects, given simultaneously as inputs, is illustrated in Fig. 8, where we used *A*_*max*_ = 0.20 for all simulations. In this figure, for brevity, only the average activity in layer L3 is presented. As it can be seen, all objects appear at least once during the simulation period. However, there is no fixed sequence; the objects emerge in an unpredictable order and with a different occurrence. Furthermore, the greater the number of objects given as input, the lower the frequency of the emerging rhythm, which decreases to approximately 20 Hz (i.e., in the beta range) when nine objects must be desynchronized together. We ascribe this reduction in frequency to an increased competition among the objects, which requires more time to be resolved. However, objects were not always recognized perfectly even when using the increased values of *A* synapses: in some occasions, especially concerning the desynchronization of eight or nine objects, one object failed to appear during the overall simulation period.

## Summary of the results and metrics

The previous figures show some typical examples of model behavior, which can be found in almost all trials, also by changing noise and the different objects in Fig. 2. A summary of all performed simulations concerning the semantic network using orthogonal objects (obtained by changing the object representations and the seed of noise, so as to obtain 20 different simulations per each case) is reported in Table 2, where we use three metrics to summarize the results: the number of successes (i.e., all objects recognized correctly at least twice), the frequency of the gamma rhythm and the time required to recognize all objects at least twice (see the legend of the table for more details). Furthermore, we tested also the dependence of the results on the strength of the inhibitory synapses, ${A}^{{L}_{2},{L}_{2}}$, and ${A}^{{L}_{3},{L}_{3}}$ (by acting on the maximum value *A*_*max*_, see the Appendix) to point out the dependence of the results on this crucial parameter. It is worth noting that the gamma frequency decreases the higher the number of objects, while the time for recognition obviously increases. Up to 7 objects can almost always be recognized (with only 1 exception with 5 objects). The percentage of successes is still high with 8 objects, and becomes problematic with 9 objects if *A*_*max*_ is too high. Increasing *A*_*max*_ increases the time required for object recognition and also reduces the gamma frequency (higher competition) but makes the solution with 9 objects less reliable.

**Table 2:** Metrics which summarize the results of 20 trials per case (obtained by changing the objects and/or the noise) concerning the semantic modality. Results with different values of the parameter *A*_*max*_ are shown for two different set of objects (with fixed and variable dimensions, respectively). We consider a success if the network recognizes each object given as input at least two times during the 1.5 s simulation window. We show the percentage of successes, the frequency of the gamma rhythm, the minimum, maximum and mean time required to have success during the 20 trails. Only in some cases with 8 or 9 objects, when the network fails to recover each objects two times, we provide the percentage of successes if each object is recovered at least one time. The pattern 1 considers a case of 9 objects with orthogonal fixed dimensions (not shown in Fig. 4). The second pattern corresponds to the case of 9 orthogonal objects with variable dimensions (i.e., case a in Fig. 4)

	% successes (2 times)	% successes (1 time)	Gamma frequency	T_min_ (s) (2 times)	T_max_ (s) (2 times)	T_mean_ (s) (2 times)
***A***_***max***_** = 0.15**						
*Pattern1: orthogonal fixed dimensions*						
4 objects	100% (20/20)	“	69.46	0.14	0.22	0.17
5 objects	100% (20/20)	“	51.22	0.22	0.57	0.36
6 objects	100% (20/20)	“	49.87	0.30	1.05	0.50
7 objects	100% (20/20)	“	38.79	0.54	1.41	0.83
8 objects	85% (17/20)	100% (20/20)	26	0.63	1.29	1.05
9 objects	80% (16/20)	100% (20/20)	21.76	0.71	1.30	1.07
*Pattern2: orthogonal variable dimensions*						
4 objects	100% (20/20)	“	69.08	0.12	0.26	0.18
5 objects	95% (19/20)	95% (19/20)	51.69	0.17	0.59	0.38
6 objects	100% (20/20)	“	50.80	0.32	1.02	0.62
7 objects	100% (20/20)	“	37.93	0.47	1.41	0.81
8 objects	75% (15/20)	95% (19/20)	27.93	0.68	1.39	1.08
9 objects	75% (15/20)	95% (19/20)	25.09	0.72	1.32	1.04
***A***_***max***_** = 0.20**						
*Pattern1: orthogonal fixed dimensions*						
4 objects	100% (20/20)	“	34.21	0.17	0.43	0.27
5 objects	100% (20/20)	“	22.93	0.36	0.68	0.43
6 objects	100% (20/20)	“	22.95	0.48	0.54	0.50
7 objects	100% (20/20)	“	21.83	0.58	0.89	0.67
8 objects	100% (20/20)	“	21.28	0.70	1.26	0.87
9 objects	15% (3/20)	70% (14/20)	19.66	0.54 (1 time)	1.41 (1 time)	1.08 (1 time)
*Pattern2: orthogonal variable dimensions*						
4 objects	100% (20/20)	“	33.51	0.17	0.56	0.31
5 objects	100% (20/20)	“	23.50	0.37	0.53	0.44
6 objects	100% (20/20)	“	22.98	0.47	0.67	0.51
7 objects	100% (20/20)	“	22.24	0.58	0.88	0.68
8 objects	65% (13/20)	100% (20/20)	21.36	0.81	1.21	0.91
9 objects	5% (2/20)	25% (5/20)	20.29	0.54 (1 time)	1.11 (1 time)	0.77 (1 time)

### Analysis of pathological conditions

**Fig. 9:**
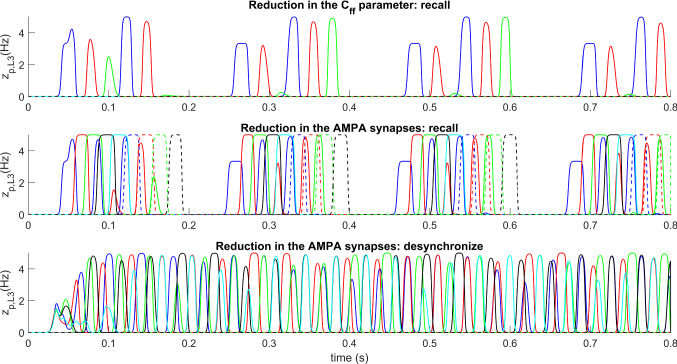
Three examples of model behavior in *pathological conditions*. In this figure, only the average spike density of the different objects in layer L3 is presented for brevity. The upper panel shows model behavior in the modality “sequence ordering memory” after presentation of the *object 1*, after a reduction of parameter *C*_*ff*_ (which represents the auto-inhibition of the fast GABAergic interneurons) to 1/4 of its normal level. This change can simulate alterations in Alzheimer disease. The second and third panels show model behavior in the modality “sequence ordering” after presentation of the *object 1*, and in the modality “semantic memory” after presentation of five objects simultaneously. These simulations have been performed after a reduction in the strength of synapses *A* to 1/4 of their original value (an alteration which can mimic that occurring in schizophrenic patients). Note that the network can neither correctly recover the sequence nor correctly desynchronize objects

Some recent experimental studies report that, in subjects affected by *Alzheimer disease*, an anomalous behavior of the neural networks related to memory (hippocampal neural networks) is linked to dysfunctions of the interneurons—in particular, the inhibitory interneurons involved in the generation of gamma oscillations (GABAfast) (Verret et al. 2012; Palop and Mucke 2016). Furthermore, some results prove that, starting from the pathological condition, correcting the activity of these neurons impacts on the state of the hippocampal network, making its activity more similar to that found in healthy subjects (Park et al. 2020). In order to analyze these conditions, we simulated the recovering of a sequence (i.e., the same situation as in Fig. 6) after a reduction of parameter *C*_*ff*_, which represents the auto-inhibition of the fast GABAergic interneurons. This parameter, as demonstrated in our previous works (Ursino et al. 2010) is closely involved in the generation of the gamma rhythm. Results, shown in the upper panel of Fig. 9 (concerning the layer L3 only) show that, after a reduction of this parameter to 1/4 of its basal value, the network becomes unable to recover a correct sequence. Just the initial two or three patterns in the list are recovered, and they occur repeatedly inside the same theta cycle.

The second test concerns schizophrenia. It has been reported in the literature that subjects suffering from this pathology exhibit alterations in the AMPA receptors (Zeppillo et al. 2020) (in the present model these may be associated with the synapses named *A*), as well as an anomalous coupling between theta and gamma rhythms in the prefrontal areas during working memory (Barr et al. 2017). To simulate a similar pathological condition, we used the network in both its modalities (“sequence ordering” and “semantic”), after a reduction in the strength of synapses *A* to 1/4 of their original value. Results shown in the mid panel of Fig. 9 demonstrate that, in this condition, the network is unable to recover a correct sequence of objects: unlike the previous case, however, now we can observe too many objects superimposed together; in particular, the first items of the sequence restarts again within the same theta cycle, superimposing with the subsequent items of the sequence in a confusing way. A similar superimposition of items is evident also in the modality “semantics” (bottom panel of Fig. 9), where we gave five objects as input to the network.

### Imagination or dreaming modality

As a last simulation, we analyzed the model behavior when the network does not receive any external input, but just a uniform noisy excitation is given to layer L1. This condition wishes to simulate a circumstance in which the subject is isolated from the environment, but can “imagine” or “dream” autonomously, by spontaneously recovering and recombining the information stored in layers L1, L2 and L3. In particular, we analyzed the network in the modality “sequence recovering”, i.e., when the synapses from L3 to L2 were previously trained to memorize a list of items.

**Fig. 10:**
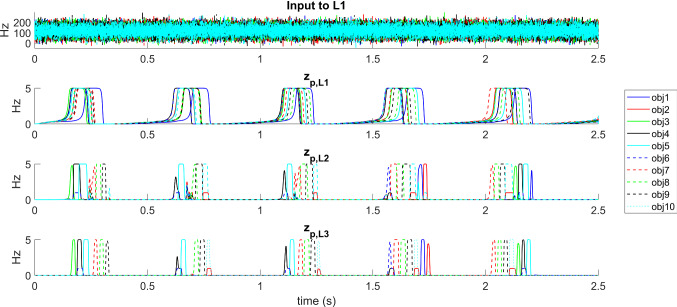
Simulation of model behavior, in the modality “sequence ordering memory”, when the network is disconnected from the external world, and layer L1 receives a uniform excitation noise (“imagination” or “dreaming” condition). In this case the network was previously trained with two alternative sequences of objects taken from Fig. 4b (sequence “1–2–3–4-5” and sequence “6–7–8–9–10”). Note that the network can autonomously recover some portions of the previously learned sequences, in a random fashion, and sometimes link together the end of one sequence with a portion of the other sequence

The simulation in Fig. 10 refers to a condition in which all pyramidal populations in layer L1 receive an input noise with uniform distribution ranging between 80 and 160. This signifies that layer L1 is disconnected from the working memory, and is “over excited” uniformly. As it can be seen from the figure, which represents a typical simulation, thanks to the presence of much noise the network can spontaneously recover several patterns in L1 (it is worth noting, however, that now the dynamics in L1 is slower, down to approximately 2 Hz). Just one of these patterns, however (generally, the first active in L1), wins the competition in L2, causing a sequence to start. Some aspects deserve attention. First, the sequence “imagined” can change from one moment to another. Second, in these simulations we used the objects in the second configurations of Fig. 4 (bottom panel), and the network was previously trained to independently learn two list of objects: “1–2–3–4-5” and “6–7–8–9–10”. Moreover, the object 6 in the second list has a portion in common with object 4 of the first list, and the object 10 in the second list has some elements in common with object 2 of the first list. We can see that sometimes the network links the two lists in L3 to form a longer hybrid list. In particular, we can observe that sometimes object 7 in the second list is evoked with a small delay after object 5 in the first list, and object 3 in the first list is evoked with a small delay after object 10 in the second list, i.e., a different list is linked at the end of the previous one. For instance, the first gamma sequence in the simulation is composed of the objects “3–4–5” + “7–8–9” (the first three items belonging to the first list, the other three to the second list) and the third gamma sequence is “4–5” + “7–8–9–10”. We can explain this behavior if we assume that the appearance of object 4 in the first list evokes a small activity for the object 6 in the second list too (since these two objects have some common features). This is not sufficient to evoke the entire object 6 in layer L3, but its partial activity favors the excitation of object7 in L2 (which is the subsequent object in the second list). As a consequence, when the first list terminates, this object is facilitated to appear. Similarly, the last sequence in the simulation consists of the objects “7–8–9–10” + “3–4–5” (the first four items belonging to the second list, the other three to the first list). In this case, the appearance of object 10 in the second list evokes a small activity for the object 2 in the first list, and the residual activity of object 2 in L3 favors the emergence of object 3 in L2.

The previous considerations can explain most of the combinations observed during our simulation. However, it is also possible that a list emerges after another in a more independent and less predictable way, as in the case of the second gamma sequence of Fig. 10 (“4–5” + “8–9–10” which is a delayed version of the third sequence lacking the object 7), or the case of the fourth gamma sequence (“6–7–8–9–10” + “1–2” which can only be explained by a random appearance of object1 at the end of the other list). These patterns are representative of what is occurring in most simulations.

Briefly, the following main conclusions can be drawn: (i) the network, deprived from any external input and disconnected from the WM layer, in the presence of a small uniform excitation and much noise, can autonomously evoke some of the learned lists; (ii) even more important, the network can link some lists together, frequently exploiting the similarity among items; (iii) in any case, it is necessary that one list terminates to allow the concatenation with another one. These aspects will be further commented in the Discussion and lines for future improvements will be given.

## Discussion

### General considerations

Working memory denotes the ability to maintain information in the brain during short time periods for immediate use: it involves the initial encoding of information, the retrieval of WM items from external cues or stimuli, and their maintenance during a delayed time until a goal is achieved. WM is crucial for many goal-directed cognitive functions and exhibits different characteristics, depending on the specific problem that has to be dealt with. Traditional models assume that WM is based either on persistent firing of some neurons in the prefrontal cortex (PFC), whose activity is maintained via recurrent positive connections until a new stimulus reset them (Funahashi et al. 1989; Compte et al. 2000) or via short-term potentiation of synaptic connections (Sandberg et al. 2003; Mongillo et al. 2008). However, data collected in the last decade evoke a much more complex scenario. First, the interaction between working memory and long term memory has become a topic of much current interest (Burgess and Hitch 2005). As argued by Burgess and Hitch (2005), models of short term memory and long term memory exhibit a crucial link, so that a complete separation among them is often not possible. Second, there is a wide consensus that WM does not only implicates the PFC, but involves several different areas, in particular the hippocampus and the parieto-occipital regions (Johnson et al. 2017). Finally, and more important, many experiments, both in rodents and humans, suggest that WM activity crucially depends on brain rhythms, in the theta, alpha and gamma bands. The role of neural oscillations in memory has become a crucial topic in recent neuroscience research.

Aim of the present work was to develop an original mathematical model to investigate the role of theta and gamma rhythms in memory. Our attention was especially focused on WM, although it is not always possible to distinguish between long term and short term memory within the present theoretical framework. Indeed, as pointed out by Manohar et al. (2019) whether a model describes short term or long term memory (hence WM, or semantic or episodic memory) depends on the duration of the synaptic changes incorporated on the Hebbian rules, i.e. on short term vs. long term potentiation, a problem that we do not explicitly address in the present model. Actually, a preponderant role of gamma-theta coupling has been demonstrated not only in studies concerning WM tasks (Lundqvist et al. 2011; Chaieb et al. 2015; Rajji et al. 2017; Tamura et al. 2017; Bahramisharif et al. 2018; Reinhart and Nguyen 2019), but also associative tasks (Köster et al. 2018), long term spatial memory (Vivekananda et al. 2021), and episodic memory (Hsieh and Ranganath 2014), thus stressing the probable presence of similar underlying mechanisms for different memory types.

Our model is based on a few basic assumptions which, although realistic, still require a future validation. What is important in the present study is the proposed architecture and the basic mechanisms incorporated, while individual details can be the subject of future improvements. Fundamentally, we demonstrated that a single multilayered structure, which makes use of neural mass models and Hebbian mechanisms, can solve different kinds of working memory problems, either involving an ordered sequence of items or segmentation of several independent items, exploiting theta and gamma oscillatory patterns. Moreover, we demonstrated that some alterations in synaptic mechanisms can potentially induce anomalies in the way memory is restored and managed, simulating pathological conditions. Finally, the free network, isolated form the environment in the presence of much superimposed noise can exhibit a dreaming or imagination behavior. All these aspects indicate that the model can represent a promising tool to investigate several fundamental problems in cognitive neuroscience, within a single theoretical framework.

At present we have not a unique indication on where the individual layers of the model can be located, but some hypotheses can be formulated on the basis of the present knowledge.

### Basic model assumptions and layers

The *Layer WM* implements the core of working memory, where the different items are initially evoked and auto-sustained and is likely located in the PFC. This region has been traditionally considered a fundamental location for the short-term goal oriented memory (Fuster and Alexander 1971). A basic assumption in our model is that the memory content is maintained thanks to a positive reverberating loop, which is reset as soon as the memory content must be updated. This idea finds some support in the literature, and can be explained via a BG-thalamus gating mechanism (Bolkan et al. 2017; Nir-Cohen et al. 2020).

*Layer L1*, where the theta rhythm originates and the items are reconstructed, could be ascribed to the hippocampus, for instance involving the lateral entorhinal cortex and CA3. There are various aspects in the literature that support this model subdivision. The hippocampus is an evident candidate for the generation of theta rhythm (Bastiaansen and Hagoort 2003; Mitchell et al. 2008), as also shown by a large literature on hippocampal theta oscillations in rats (Vinogradova 1995; Buzsáki 2002, 2005). The medial PFC is bilaterally connected to the hippocampus through the perirhinal and lateral entorhinal cortex. Experiments in which neural activity is simultaneously recorded in the rodent mPFC and in the hippocampus have demonstrated that mPFC neuronal spiking occurs at a specific phase of hippocampal theta oscillations (Hyman et al. 2003; Siapas et al. 2005; Jones and Wilson 2005; Gordon 2011; Kim et al. 2011) that this phase relationship is especially prominent after learning (Kim et al. 2011), and that PFC spiking activity is best phase-locked to hippocampal theta oscillations occurring in approximately the past 50 ms (Siapas et al. 2005). The latter results agree with the patterns shown in Fig. 5, where, in the “sequence ordering” modality, the network exhibits a theta phase-lock between the WM and L1 after learning: the theta rhythm originates in L1 and is transmitted back to WM with a 40–50 ms time delay. A bidirectional flow of information between the PFC and the hippocampus has been recently observed in context-driven memory trials (Place et al. 2016). Moreover, the hippocampus CA3 region has been traditionally considered an ideal place for auto associative completion (Marr 1971; Treves and Rolls 1994; Hasselmo et al. 1995) due to the presence of abundant recurrent collaterals (Li et al. 1994) and Hebbian synaptic modification (Bliss and Collingridge 1993). Of course, alternative hypotheses can also be viable, and can be tested in different future models. For instance, theta rhythms may be generated in multiple sources of the brain, not just in the hippocampus, and are known to propagate through the Default Mode Network (see Hsieh and Ranganath 2014). The latter observation however does not contradict our simulations: in our model theta rhythm actually propagates from L1 to downstream layers and, likely, spreads toward other brain areas too (not included in the model), where the evoked memory patterns are utilized and manipulated (but see also Zhang et al. 2020; Yuan et al. 2021 for recent studies on the subject).

A particular aspect of our model is that the theta rhythm is generated directly within the L1 auto associative layer as a consequence of strong Hebbian learning. A more traditional hypothesis is that the theta rhythm is generated in the septum, which possibly acts as a pacemaker for theta activity (Pignatelli et al. 2012) and from there is transmitted to hippocampal neurons (Roux and Uhlhaas 2014). However, more recent data have shown that oscillations in the theta range can be recorded in hippocampal preparations in vitro, thus confirming that the hippocampus itself can act as a theta oscillator (Cataldi and Vigliotti 2018).

Basically, in our model two conditions are necessary to generate a clear theta rhythm: a moderate external input (in our case coming from the WM layer) and excitatory auto-associative synapses induced by Hebbian learning. As consequence, a group of pyramidal neurons in the same item is maximally excited, and strongly excites GABAergic inhibitory interneurons with slow synapse dynamics. The latter, in sequence, inhibits pyramidal neurons causing a decrease in the overall activity to zero. This model mechanism agrees with the ideas (Roux and Uhlhaas 2014; Colgin 2016) that “In the hippocampus, theta oscillations are generated by an interplay of glutamatergic and GABAergic neurons” and represents an important testable hypothesis of our model, requiring further verification.

However, an important characteristic of our theoretical framework is that theta rhythms are generated only when the network works in the modality “sequence ordering”. In the “semantic memory” modality the theta rhythm is practically absent. Of course, this implies the existence of two alternative circuits linking WM and L1: the first, with weaker connections, devoted to temporal sequencing, based on a robust theta, and the other, with stronger connections, devoted to the segmentation of items and their semantics recognition without any assigned order. This is a further model prediction requiring future analysis. Cohen (2011) suggests that the relationship between theta activity and memory encoding might depend on connectivity between the hippocampus and PFC. The possibility of a different connectivity weight between the PFC and two alternative pre-processing hippocampal pathways (named “what” and “where” by the authors) is discussed in Preston and Eichenbaum (2013).

Several works support the existence of alternative circuits in the PFC-hippocampus pathways functionally differentiated along its dorsoventral axis and the ventral (anterior) hippocampal formation (Moser and Moser 1998; Hoge and Kesner 2007; Farovik et al. 2010). Furthermore, recent data suggest that the theta rhythm is prominent only during WM problems which involve temporal order, but plays a less important role in tasks which do not require any temporal sequencing (Roux and Uhlhaas 2014; Hsieh et al. 2011; Heusser et al. 2016).

The *layer L2* in our model, probably located in the hippocampus CA3 area, is essential for the production of the gamma rhythm, involving GABAergic interneurons with faster kinetics. This modeling requisite is confirmed by several studies which underline the critical role of fast-spiking parvalbumin (FS) interneurons in the emergence of cortical gamma activity (Bartos et al. 2007; Cardin et al. 2009; Whittington et al. 2011). In the present model, the gamma code is generated downstream of the theta rhythm (specifically in layers L2 and L3), and involves both Hebbian and anti-Hebbian mechanisms. The Hebbian mechanism produces strong glutamatergic synapses from pyramidal neurons in an object to fast GABAergic interneurons in the same object, generating a gamma rhythm and solving the binding problem. Anti Hebbian mechanisms are used to create much more rapid connections from pyramidal neurons in an object to fast inhibitory interneurons in other objects, to solve the segmentation problem.

An important question concerns the nature of the rapid synapses (named *A* in the model) working with a very fast time scale: they might involve very fast AMPA receptors or more probably gap junctions. Schmitz et al. (2001) provided evidence that axons of hippocampal principal cells are electrically coupled, and can represent a mechanism for very fast electrical communication. The idea that principal cells in the hippocampus establish electrical synapses with each other, implicated in network oscillations and synchronization, is further supported by additional recent studies (Molchanova et al. 2016; Ixmatlahua et al. 2020). Furthermore, recent experiments suggest that the strength of gap junctions can be modified in an activity-dependent manner, similar to that of chemical synapses (Cachope et al. 2007; Turecek et al. 2014; Wang et al. 2015). Pernelle et al. (2018), using a computational model, demonstrated that gap-junction plasticity can play a role to regulate oscillations and transmit information in a network of inhibitory and excitatory neurons. These results confirm several assumptions of the present model but, of course, require further analysis.

However, we must mention a further alternative hypothesis for fast desynchronization in L2 and L3. In fact, Kwon et al. (2018) showed that Schaffer collateral from CA3 to CA1 innervate both excitatory pyramidal cells and inhibitory interneurons, but with different connectivity rules. Hence, it is also possible that a very fast inhibition, to desynchronize objects, is provided by a feedforward link from a previous layer to inhibitory interneurons in a downstream layer. This may represent an alternative desynchronization mechanism which requires attention in future work.

Finally, a feedback external loop has been introduced in L2, to synchronize the gamma rhythm with the on phase of the theta rhythm originating in L1, as observed in hippocampus place cells. Evidence for a similar loop can be found in the literature, in particular involving the so-called Papez’s circuit which involves the hippocampal formation, the mammillary bodies and the anterior thalamus. There is large evidence that the theta rhythm resonates throughout this circuit and that this circuit can have a role for coordinating hippocampo-cortical activity and to control mnemonic functions (Vertes et al. 2001; Dillingham et al. 2021). A further similar mechanism may involve the medial septum, which acts through a dishinibition of the hippocampus, i.e., a mechanisms very similar to the one proposed in the present work (Kocsis and Kaminski 2006; Salib et al. 2019).

*Layer L3* in our model has two functions: to further improve segmentation and, in the sequence ordering mode, to produce a sequence of items via Hebbian feedback connections. It is not easy and probably not unique to determine the relative location of layers L2 and L3. In the classic model by Lisman et al. (2005), where however the position of the hetero- and auto-association networks are inverted compared with ours, a similar mechanism was ascribed to the dentate gyrus and CA3. According to an hypothesis developed in Yamaguchi et al. (2007) (see Fig. 1 in that paper), a similar mechanism may involve CA3, CA1 and the superficial layers of the entorhinal cortex. In our opinion CA1 is a good candidate for the sequential order modality. In fact, the existence of feedback from CA1 to CA3 neurons is well documented, via the participation of the entorhinal cortex (Eichenbaum 2017). Sandler et al. (2015) demonstrated the presence of a Granger causality not only from CA3 to CA1 (feedforward) but also from CA1 to CA3 (feedback). Hoge and Kesner (2007) observed that rats with CA1 lesions displayed a profound deficit in remembering the order of the visual object presentations, suggesting that the CA1 is critical for processing temporal information. Other studies suggesting that CA1 area is selectively required for temporal coding are summarized in Mankin et al. (2012).

### Model working in alternative conditions

An important aspect of our model is that it can be used to investigate, although at a preliminary stage, additional important neurocognitive problems directly connected with memory. In particular, we focused attention on two main questions: the role of gamma rhythms during imagination and dreaming, and the alterations in the memory encoding and retrieval occurring as a consequence of synaptic alterations (mimicking pathological conditions such as Alzheimer or schizophrenia).

The present network, isolated from the external world and stimulated with noise in the L1 layer, can randomly replay some of the sequences previously memorized. Moreover, if some of these sequences exhibit a few superimposed features (i.e., items are not completely orthogonal) they can be recombined in a new and creative manner, so that the terminal portion of a sequence is linked to portions of another one. This result opens interesting perspectives for comprehension of some fascinating neurocognitive problems, such as those involved in dreaming or imagination. For instance, place cells in the rat hippocampus not only fire during an experience, but also later ‘replay’ sequences in a similar order or in reverse order during sleep (Skaggs and McNaughton 1996; Dragoi and Buzsáki 2006; Foster and Wilson 2007) or can even construct never-experienced new path-sequences (Gupta et al. 2010).

Finally, with the present model we simulated two different alterations in model synapses, which can have implications in neurological disorders. Indeed, results in the literature suggest that theta-gamma coupling is impaired in schizophrenic subjects, and this modification can determine WM dysfunctions (Cho et al. 2006; Basar-Eroglu et al. 2007; Barr et al. 2010, 2017; Berger et al. 2016). A common hypothesis is that these alterations depend on a deficiency in GABAergic parvalbumin interneurons (Lewis et al. 2012): these findings suggest a new model of cortical dysfunction in schizophrenia in which inhibition is decreased. Accordingly, we tested two different dysfunctions concerning fast inhibitory interneurons in our model. First, we decreased the strength of the synapses *A*, that target into fast interneurons and are essential for correct desynchronization of items, thus producing a smaller inhibition in the network compared with the normal case. The consequence is that the model, in the “sequence ordering” modality, becomes unable to recover a sequence correctly, producing a complex and confused mixing of various items (Fig. 9 middle panel) with evident alterations in sequential WM; in the “semantic” modality, the network cannot segment items correctly (Fig. 9 bottom panel), thus producing a confused superimposition of features in different objects. Although still at an oversimplified stage, these preliminary simulations can provide some cues on the origin of some positive symptoms in schizophrenia, such as hallucinations, incoherent thinking, distortions. Another possible alteration simulated with the model concerns a reduction of parameter *C*_*ff*_, which represents the auto-inhibition of the fast GABAergic interneurons. After this change, the network loses the capacity to evoke a sufficient number of items (Fig. 9 upper panel when only three items are recalled), i.e., it works in a condition of memory deficiency. This behavior can simulate some aspects of Alzheimer disease. Various recent studies underline the existence of deficits in the GABA–ergic interneurons in the Alzheimer too (Xu et al. 2020).

Of course more sophisticate combinations of parameter changes can be performed in future works, with a more detailed comparison with the neurobiology, to emphasize the possible use of the model in the comprehension of neurological deficits.

### Testable predictions

Our model makes use of various hypotheses which, although justified by the present knowledge on the prefrontal cortex-hippocampus interactions, still need a validation. Based on these hypotheses, the results lead to some testable predictions, which can be verified in future studies.

(i) the model assumes the presence of very fast inhibitory mechanisms, working on a time scale smaller than gamma, essential to desynchronize objects. In the “semantic modality” the model can segment three or four objects simultaneously quite easily, using a phase desynchronization in the gamma band. This agrees with the classic number of items assumed in WM. A weakening of this mechanism is associated with poor segmentation and confusion among objects; an increase in this mechanisms can allow the solution of more complex segmentation problems (involving up to nine objects). It is possible that a control in the strength of synapses incorporates a sort of attentional mechanism: the higher the number of items, the stronger the attention devoted to that particular task. The synapse arrangement of these mechanisms is anti-Hebbian. A test should study the presence of ultra-fast inhibition, the effect of its impairment and possible the arrangement of these synapses (anti-Hebbian).

(ii) The theta rhythm plays a different role in sequence ordering problems and in scene segmentation problems. Different WM tasks would exhibit a different dependence on this rhythm. A test can analyze the dependence of the theta power on the role of temporal ordering in the assigned task.

(iii) The model assumes that brain rhythms in the hippocampus are affected by Hebbian (and anti-Hebbian) mechanisms, hence by past experience and by the process of object storage. Hence, the storage of objects can modify these rhythms, by strengthening or weakening them. An alternative hypothesis is that these rhythms are intrinsic (i.e., independent of previous memorization). These alternative hypotheses can be checked by studying the dependence of rhythm power and frequency on the task and the storage requirements.

(iv) Model results show that the frequency of the gamma rhythm decreases when a greater number of objects must be simultaneously segmented in a given semantic problem. The dependence of frequency on the task complexity can be tested.

(v) The same structure (in our case the pre-frontal cortex – hippocampus complex) can solve different memory problems in different segregated circuits, as a consequence of a different learning of feedback synapses and different potentiation of feedforward synapses.

### Future study

In the present work we made use of a neural mass model which, although largely used in practice, is of course a simplification of the real dynamical behavior of spiking neurons. A fundamental problem, which requires detailed analysis in future work, is the relationship between the sigmoidal formulation in the NMM and a biophysical description of spiking neurons. Briefly, the capacity to produce oscillations, their frequency content and amplitude, and transmissibility in a NMM crucially depend on the position of the various populations on the sigmoidal relationship. It will be worthwhile in future work to simulate the same connections and possibly similar waves with populations of spiking neurons to test similarities and differences. Spiking neurons offer the advantage of a greater physiological reliability and likely the possibility of more complex pieces of behavior, at the cost of increased computational time and, above all, of a greater difficulty to summarize the results into a simple synthetic frame. The possibility to work out with multiscale models, which move from the neuronal level to the population levels and back to neurons will be an essential subject of research in future studies.

In this work we demonstrated that the model can solve different problems in WM using brain rhythms and a four layer structure. However, this general framework is quite flexible and can be modified in future work to meet additional data and accommodate new ideas. For instance, a frequent idea is that a gamma rhythm is generated in the brain upstream of the prefrontal region (and not in downstream layers as in our model) and transmitted to the PFC from the input connections (for instance from occipital visual regions to frontal regions, which in turn exhibit a top-down influence, (see Fries 2015). However, this choice would require a modification of the present auto-associative net, since in this case auto-association would necessitate that the gamma cycles are synchronized before the involvement of a theta rhythm.

A problem in our model is that, if a given object or item appears entirely in two different sequences, the model has no mechanism to choose whether to continue on one path or on another. For this reason, we never simulated such a condition: all the objects memorized in this paper, although not orthogonal, exhibit only a limited percentage of common features (less than 20%). As suggested by Alexander et al. (2020), the possibility to disambiguate multiple overlapping spatiotemporal trajectories requires the presence of more sophisticate cells, which differentially code the context of prior or future behavior. This important question may be the subject of future model improvements. In particular, the possibility to manage overlapping trajectories can strongly improve the model’s ability to simulate dreaming, imagination and pathological behavior.

## Figures and Tables

**Fig. 11:**
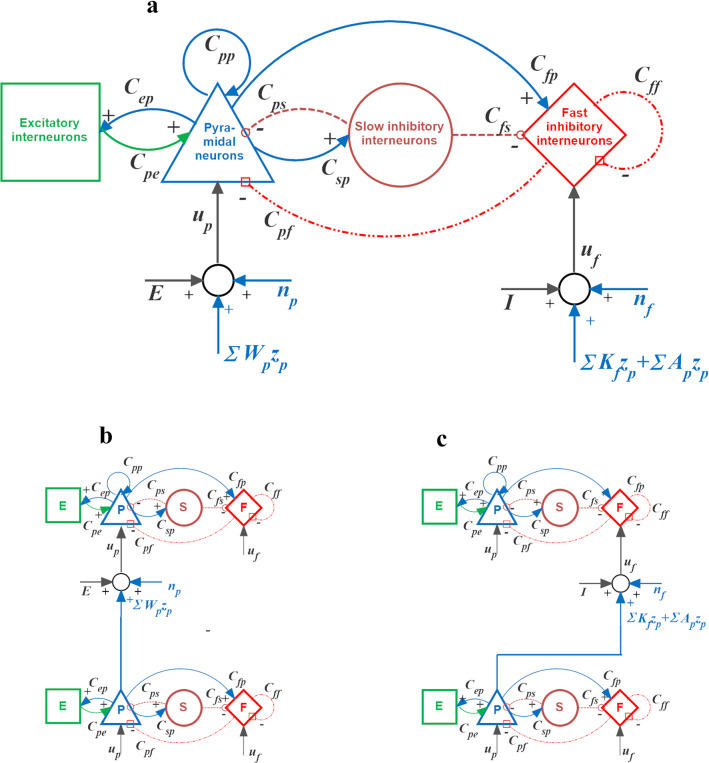
*Panel A*: Scheme of the neural mass model simulating the dynamics in a single column. Blue continuous lines with arrows indicate glutamatergic excitatory synapses, red dash-dotted lines with open squares indicate GABAergic faster inhibitory synapses, while brown dotted lines with open circles indicate GABAergic slower inhibitory synapses. Symbols *C*_*ij*_ denote the synaptic contacts among the neural populations, where the first subscript and the second subscript designate the post-synaptic population and pre-synaptic population, respectively. *u*_*p*_ and *u*_*f*_ represent inputs to the pyramidal neuron population and to the fast inhibitory interneuron population, respectively. These inputs can come from the external environment (*E* and *I* respectively), from noise (*n*_*p*_ and *n*_*f*_, respectively) or from synapses from pyramidal neurons in other ROIs. *Panel B*: an example of excitatory connections between two ROIs, via a direct link from the pyramidal neurons of the source ROI to the pyramidal neurons of the target ROI. *Panel C*: an example of a bi-synaptic inhibitory connection, from the pyramidal neurons of the source ROI to the fast inhibitory interneurons of the target ROI (which, in turn, inhibits pyramidal neurons in the target ROI). In the present model the latter connection may be either of type *K* (with glutamatergic dynamics) or type *A* (with almost instantaneous dynamics)

## Data Availability

Not applicable.

## Appendix: Equations and parameters

In this appendix, we present the model’s equations, the training rules and all related parameters.

Figure 11 summarized the main relationships among quantities, in a more complex form compared with Fig. 1 of the main text.

### Synapses

All synapses in the model are described by the following second order differential equation:

1 $$\begin{array}{c}\frac{{d}^{2}{y}_{n}\left(t\right)}{d{t}^{2}}= \frac{{G}_{n}}{{\tau }_{n}}{z}_{n}\left(t\right)- \frac{2}{{\tau }_{n}}\frac{d{y}_{n}\left(t\right)}{dt}- \frac{{y}_{n}\left(t\right)}{{\tau }_{n}^{2}} \end{array}$$

where ${G}_{n}$ is the gain, ${\tau }_{n}$ is the time constant and *z*_*n*_ is the input to the synapse, i.e. the presynaptic spike density. The subscript *n* is a generic one; it stands for either *p*, *e*, *s*, or *f*, depending on the neural population the equation is referring to: *p* for pyramidal neurons, *e* for excitatory interneurons, *s* for slow inhibitory interneurons, *f* for fast inhibitory interneurons. All second order differential equations of type A1 are equivalent to the two first order differential equations that follow.

2 $$\begin{array}{l}\left\{\begin{array}{c}\frac{d{y}_{n}\left(t\right)}{dt}={x}_{n}\left(t\right)\\ \frac{d{x}_{n}\left(t\right)}{dt}= \frac{{G}_{n}}{{\tau }_{n}}{z}_{n}\left(t\right)- \frac{2}{{\tau }_{n}}{x}_{n}\left(t\right)- \frac{{y}_{n}\left(t\right)}{{\tau }_{n}^{2}}\end{array} \right.\end{array}$$

### Model of a single cortical column

For each neuronal population, we first computed the mean membrane potential *v(t)*, which is influenced by synaptic connections. Then we computed the average firing rate of the population, *z(t)*, through a sigmoidal activation function, *S(v(t))*. Finally, a normalized post-synaptic potential, *y(t)*, can be computed using the Equations (A2); the latter must be multiplied by the synaptic weight to determine the actual contribution to the post-synaptic membrane potential.

Both pyramidal neurons and fast inhibitory interneurons can receive an external input—labeled *u*_*p*_ and *u*_*f*_, respectively. These are random variables with normal distribution, mean value *m*_*p*_ (or *m*_*f*_) and standard deviation *σ*_*p*_ (or *σ*_*f*_). Both reach the target population through an excitatory synapse. In the case of *u*_*p*_, we implemented a common mathematical procedure to reduce the number of differential equations (and therefore, the number of state variables). Specifically, we processed *u*_*p*_ through the excitatory synapse that goes from excitatory interneurons to pyramidal neurons (see Eqs. (A4) below), instead of processing the input separately. The other external input, *u*_*f*_, reaches its target population through a dedicated synapse (Eqs. (A6)).

The membrane potential of pyramidal neurons and fast inhibitory interneurons is also influenced by long-range synapses, which connect different cortical columns. Such contributions are labelled *E*(*t*) and *I*(*t*) and will be discussed in the next paragraph.

#### Equations for all populations

Pyramidal neurons:

3′ $$\begin{array}{l}\left\{\begin{array}{c}\begin{array}{c}{v}_{p}(t)={C}_{pe}{y}_{e}\left(t\right)+{C}_{pp}{y}_{p}\left(t\right)- {C}_{ps}{y}_{s}\left(t\right)- {C}_{pf}{y}_{f}\left(t\right)+E\left(t\right)\\ S\left({v}_{p}(t)\right)={z}_{p}\left(t\right)= \frac{2{e}_{0}}{1+ {e}^{r({s}_{0} - {v}_{p})}}\\ \frac{d{y}_{p}\left(t\right)}{dt}={x}_{p}\left(t\right)\end{array}\\ \frac{d{x}_{p}\left(t\right)}{dt}= \frac{{G}_{e}}{{\tau }_{e}}{z}_{p}\left(t\right)- \frac{2}{{\tau }_{e}}{x}_{p}\left(t\right)- \frac{{y}_{p}\left(t\right)}{{\tau }_{e}^{2}}\end{array}\right. \end{array}$$

It is worth noting that we assumed ${G}_{p}={G}_{e}$ and ${\tau }_{p}={\tau }_{e}$ in Eq. (3’), since both pyramidal neurons and excitatory interneurons exploit the same type of glutamatergic synapse. Moreover, it is worth noting that the self-loop ${C}_{pp}$ of pyramidal neurons has been used only in the WM layer, and is set to zero in the other layers. Hence, we can write.

3'' $$\begin{aligned}\text{In layers L1, L2 and L3:}\quad {C}_{pp}=0\\&\text{In layer WM}\quad C_{{pp}} \left( {\text{t}} \right) = \begin{array}{*{20}l} {\hat{C}_{{pp}} ,~~~~{\text{if}}~~~m_{p} \left( t \right) = 0~~~~~{\text{maintainance period}}}~ \\ {0, ,~~~~{\text{if}}~~~m_{p} \left( t \right) \ne 0~~~~~{\text{reset period}}} \\ \end{array}\end{aligned}$$

Excitatory interneurons:

4 $$\begin{array}{c}\left\{\begin{array}{c}\begin{array}{l}{v}_{e}(t)={C}_{ep}{y}_{p}\left(t\right)\\ {S\left({v}_{e}(t)\right)=z}_{e}\left(t\right)= \frac{2{e}_{0}}{1+ {e}^{r({s}_{0} - {v}_{e})}}\\ \frac{d{y}_{e}\left(t\right)}{dt}={x}_{e}\left(t\right)\end{array}\\ \frac{d{x}_{e}\left(t\right)}{dt}= \frac{{G}_{e}}{{\tau }_{e}}\left({z}_{e}\left(t\right)+ \frac{{u}_{p}}{{C}_{pe}}\right)- \frac{2}{{\tau }_{e}}{x}_{e}\left(t\right)- \frac{{y}_{e}\left(t\right)}{{\tau }_{e}^{2}}\end{array}\right. \end{array}$$

Slow inhibitory interneurons:

5 $$\begin{array}{c}\left\{\begin{array}{l}\begin{array}{c}{v}_{s}(t)={C}_{sp}{y}_{p}(t)\\ S\left({v}_{s}(t)\right)={z}_{s}\left(t\right)= \frac{2{e}_{0}}{1+ {e}^{r({s}_{0} - {v}_{s})}}\\ \frac{d{y}_{s}\left(t\right)}{dt}={x}_{s}\left(t\right)\end{array}\\ \frac{d{x}_{s}\left(t\right)}{dt}= \frac{{G}_{s}}{{\tau }_{s}}{z}_{s}\left(t\right)- \frac{2}{{\tau }_{s}}{x}_{s}\left(t\right)- \frac{{y}_{s}\left(t\right)}{{\tau }_{s}^{2}}\end{array}\right. \end{array}$$

Fast inhibitory interneurons:

6 $$\begin{array}{c}\left\{\begin{array}{l}\begin{array}{c}{v}_{f}(t)={C}_{fp}{y}_{p}\left(t\right)- {C}_{fs}{y}_{s}\left(t\right)- {C}_{ff}{y}_{f}\left(t\right)+{y}_{l}\left(t\right)+I(t)\\ S\left({v}_{f}(t)\right)={z}_{f}\left(t\right)= \frac{2{e}_{0}}{1+ {e}^{r({s}_{0} - {v}_{f}(t))}}\\ \frac{d{y}_{f}\left(t\right)}{dt}={x}_{f}\left(t\right)\end{array}\\ \frac{d{x}_{f}\left(t\right)}{dt}= \frac{{G}_{f}}{{\tau }_{f}}{z}_{f}\left(t\right)- \frac{2}{{\tau }_{f}}{x}_{f}\left(t\right)- \frac{{y}_{f}\left(t\right)}{{\tau }_{f}^{2}}\\ \frac{d{y}_{l}\left(t\right)}{dt}={x}_{l}\left(t\right)\\ \frac{d{x}_{l}\left(t\right)}{dt}= \frac{{G}_{e}}{{\tau }_{e}}{u}_{f}\left(t\right)- \frac{2}{{\tau }_{e}}{x}_{l}\left(t\right)- \frac{{y}_{l}\left(t\right)}{{\tau }_{e}^{2}}\end{array}\right. \end{array}$$

where the subscript *l* is used to represent the quantities in the additional synapse, introduced to describe the effect of the input *u*_*f*_ via glutamatergic dynamics. All related parameters’ values are listed in the Table 3.

**Table 3:** Parameters describing the dynamics of the populations within a cortical column

Synapses				Fuction S(*v(t)*)	
G_e_ (mV)	5.17	τ_e_ (ms)	7.7	e_0_ (Hz)	5
G_s_ (mV)	4.45	τ_s_ (ms)	34	r (mV^−1^)	0.7
G_f_ (mV)	57.1	τ_f_ (ms)	6.8	s_0_ (mV)	10

Inputs		Intra-column connections		C_pp_	300
m_p_ (Hz)	See text	C_ep_	31.7	C_fp_	66.9
m_f_ (Hz)	See text	C_pe_	17.3	C_fs_	100
σ_p_^2^ (s^−2^)	5	C_sp_	51.9	C_pf_	16
σ_f_^2^ (s^−2^)	5	C_ps_	100	C_ff_	18

### Long range connections

As previously mentioned, long-range synapses connect two different cortical columns, either belonging to the same layer or to different layers. These contributions appeared in the previous equations as the variables *E*(*t*) and *I*(*t*) (Eqs. (3’) and (6)), which stand for long-range Excitation and long-range Inhibition, respectively. For a more detailed qualitative description of the synaptic architecture (structure of feedforward and feedback long-range connections) we refer to the main text of the present article.

In the following, we will use subscripts (*i* or *j*) to denote the position of a cortical column within a layer, and superscripts (either *WM, L*_*1*_*, L*_*2*_ or *L*_*3*_) to represent a layer. It is worth noting that, although in Fig. 4 cortical columns are represented in a two-dimensional lattice for clarity, in the following we will assume that this two-dimensional structure is converted into a vector. Hence, just one subscript is used to represent a column, while we need two subscripts to represent synapses linking two columns. Generally, the first subscript (or superscript) will be used to represent the post-synaptic column (or the post-synaptic layer) whereas the second subscript (or superscript) will represent the pre-synaptic column (or the pre-synaptic layer). For instance, the symbol ${{W}_{ij}^{L1,WM}}$ in the following represents an excitatory synapse from a pre-synaptic neuron at position *j* in layer *WM* to a post-synaptic neuron at position *i* in layer *L1*.

Briefly, *WM* receives an excitatory feedback signal from *L1*, through the synapses ${W}^{WM,{L}_{1}}$. L1 receives information on the external input from WM through ${W}^{{L}_{1},WM}$, and implements an auto-associative network through the lateral synapses ${W}^{{L}_{1},{L}_{1}}$. L2 receives an input from L1 through ${W}^{{L}_{2},{L}_{1}}$, and can further receive a feedback from L3 through ${W}^{{L}_{2},{L}_{3}}$ (this term implements the hetero-associative network, and is absent when the model is operating in the “semantic working memory” modality), and is subjected to synchronizing and desynchronizing lateral synapses (${K}^{{L}_{2},{L}_{2}}$ and ${A}^{{L}_{2},{L}_{2}}$). L3 also has synchronizing and desynchronizing lateral synapses (${K}^{{L}_{3},{L}_{3}}$ and ${A}^{{L}_{3},{L}_{3}}$). Moreover, it receives an input from L2 through the synapses ${W}^{{L}_{3},{L}_{2}}$. Finally, it is worth noting that the values of the synapses ${W}^{WM,{L}_{1}}$ and ${W}^{{L}_{1},WM}$ are higher in the modality “semantic memory” compared with the other modality.

For each layer, *E*(*t*) and *I*(*t*) are therefore calculated as follows. The subscripts *i, j* = 1, …, 400 identify a single cortical column within the specified layer.

WM:

7 $$\begin{array}{c}\begin{array}{c}{E}_{i}^{WM}\left(t\right)= {\sum }_{j}{W}_{ij}^{WM,{L}_{1}}\cdot {y}_{p,j}^{{L}_{1}}\left(t\right)\\ {I}_{i}^{WM}\left(t\right)=0\end{array}\end{array}$$

L1:

8 $$\begin{array}{c}\begin{array}{c}{E}_{i}^{{L}_{1}}\left(t\right)= \sum_{j}{W}_{ij}^{{L}_{1},WM}\cdot {y}_{p,j}^{WM}\left(t\right)+{\sum }_{j}{W}_{ij}^{{L}_{1},{L}_{1}}\cdot {y}_{p,j}^{{L}_{1}}\left(t\right)\\ {I}_{i}^{{L}_{1}}\left(t\right)=0\end{array}\end{array}$$

L2:

9 $$\begin{aligned}{E}_{i}^{{L}_{2}}\left(t\right)&= {\sum }_{j}{W}_{ij}^{{L}_{2},{L}_{1}}\cdot {y}_{p,j}^{{L}_{1}}\left(t\right)+{\sum }_{j}{W}_{ij}^{{L}_{2},{L}_{3}}\cdot {y}_{p,j}^{{L}_{3}}\left(t\right)\\ {I}_{i}^{{L}_{2}}\left(t\right)&= {\sum }_{j}{K}_{ij}^{{L}_{2},{L}_{2}}\cdot {y}_{p,j}^{{L}_{2}}\left(t\right)+{\sum }_{j}{A}_{ij}^{{L}_{2},{L}_{2}}\cdot {z}_{p,j}^{{L}_{2}}\left(t\right)+Inhibitor\\ Inhibitor&=R\cdot {\left(T-{\sum }_{j}{z}_{p,j}^{{L}_{1}}\right)}^{+}\end{aligned}$$

L3:

10 $$\begin{aligned}{E}_{i}^{{L}_{3}}\left(t\right)&= {\sum }_{j}{W}_{ij}^{{L}_{3},{L}_{2}}\cdot {y}_{p,j}^{{L}_{2}}\left(t\right)\\ {I}_{i}^{{L}_{3}}\left(t\right)&= {\sum }_{j}{K}_{ij}^{{L}_{3},{L}_{3}}\cdot {y}_{p,j}^{{L}_{3}}\left(t\right)+{\sum }_{j}{A}_{ij}^{{L}_{3},{L}_{3}}\cdot {z}_{p,j}^{{L}_{3}}\left(t\right)\end{aligned}$$

The Eq. (9), which refer to L2, contain the definition of the term $Inhibitor$, where the function ()^+^ stands for the operator “positive part”. This term receives the overall activity from layer L1 and compares this activity with a given threshold, *T*. If the global activity in L1 is below threshold, the inhibitor silences all cortical columns in layer L2 by acting on the inputs of the fast inhibitory interneurons. This inhibition is then retracted as soon as the global activity in L1 rises above threshold. In other words, $Inhibitor$ is the term we used to implement the modulation of L2 (and subsequently L3) by L1, to facilitate the coupling between theta and gamma rhythms.

The values of parameters pertaining to the equations above, as well as the values of fixed synapses, are presented in the Table 4.

**Table 4:** Parameters describing the long range synapses among layers. All these arrays are diagonal in type

${W}^{{L}_{1},WM}$*	100	${W}^{{L}_{2,}{L}_{1}}$	120
${W}^{{L}_{1},WM}$**	300	${W}^{{L}_{3}{,L}_{2}}$	186
${W}^{WM,{L}_{1}}$*	100	T	20
${W}^{WM,{L}_{1}}$**	300	R	1000

^*^If the network is operating in the “sequence-ordering working memory” modality

^**^ If the network is operating in the “semantic working memory” modality

### Training rules

We used Hebbian and anti-Hebbian rules to train the synapses. For a more detailed description of the training procedures, we refer to the section “Training the network” of the present article.

#### Synapses within layer L1

##### Hebb rule (long term potentiation)

11 $$\begin{array}{c}\begin{array}{c}{\Delta W}_{ij}^{{L}_{1},{L}_{1}}\left(t\right)={\gamma }_{W}{\left(\frac{{z}_{p,i}^{{L}_{1}}\left(t\right)}{2{e}_{0}}-{\theta }_{low1}\right)}^{+}{\left(\frac{{z}_{p,j}^{{L}_{1}}\left(t\right)}{2{e}_{0}}-{\theta }_{low1}\right)}^{+}\left({W}_{max}^{{L}_{1},{L}_{1}}{-W}_{ij}^{{L}_{1},{L}_{1}}\left(t\right)\right)\\ {W}_{ij}^{{L}_{1},{L}_{1}}\left(t+{T}_{s}\right)= {W}_{ij}^{{L}_{1},{L}_{1}}\left(t\right)+ {\Delta W}_{ij}^{{L}_{1},{L}_{1}}\left(t\right)\end{array} , i\ne j\end{array}$$

where ${\gamma }_{W}$ represents a learning factor, 2*e*_0_ is the maximum firing rate (hence, all activities are normalized to the maximum), ()^+^ is the function “positive part”. It is worth noting that the pre-synaptic and post-synaptic activities are compared with a threshold θ_l*ow*1_, and the synapse is increased only if both activities are above threshold. This means that ${\Delta W}_{ij}^{{L}_{1},{L}_{1}}\left(t\right)$ can only be positive, i.e. the synaptic weights cannot decrease over time and the network is unable to forget—a limitation that could be overcome in future projects. Finally, the last term in Eq. (11) means that the synapse cannot overcome a maximum saturation value, ${W}_{max}^{{L}_{1},{L}_{1}}$, and so that the overall learning rate decreases approaching saturation.

##### Normalization to a maximum

After application of the Hebb rule, we checked that the sum of the synapses entering into a cortical column cannot overcome a global maximum, named ${W}_{maxsum}$. This allows objects with different dimension to receive the same amount of global excitation; moreover, this choice corresponds to a physiological limitation of the overall neurotransmitter available. We have

12 $$\begin{aligned}{S}_{i}^{W}&=\sum_{j}{W}_{ij}^{{L}_{1},{L}_{1}}. \\ I\!f\; {S}_{i}^{W}&> {W}_{maxsum} \Rightarrow {W}_{ij}^{{L}_{1},{L}_{1}}\leftarrow {W}_{ij}^{{L}_{1},{L}_{1}}\cdot \frac{{W}_{maxsum} }{{S}_{i}^{W}} \end{aligned}$$

#### Synapses within layers L2 and L3

The procedures to train the lateral connections within these two layers are exactly the same, however by considering that the post-synaptic population consists of fast inhibitory interneurons. Therefore, for the sake of brevity, we present only the equations pertaining to the training of ${K}^{{L}_{2},{L}_{2}}$ and ${A}^{{L}_{2},{L}_{2}}$, Analogous equations can be written as to ${K}^{{L}_{3},{L}_{3}}$ and ${A}^{{L}_{3},{L}_{3}}$ if superscripts are properly changed.

13 $$\begin{array}{c}\begin{array}{c}{\Delta K}_{ij}^{{L}_{2},{L}_{2}}\left(t\right)={\gamma }_{K}{\left(\frac{{z}_{f,i}^{{L}_{2}}\left(t\right)}{2{e}_{0}}-{\theta }_{low2}\right)}^{+}{\left(\frac{{z}_{p,j}^{{L}_{2}}\left(t\right)}{2{e}_{0}}-{\theta }_{low2}\right)}^{+}\left({K}_{max}^{{L}_{2},{L}_{2}}{- K}_{ij}^{{L}_{2},{L}_{2}}\left(t\right)\right)\\ {K}_{ij}^{{L}_{2},{L}_{2}}\left(t+{T}_{s}\right)= {K}_{ij}^{{L}_{2},{L}_{2}}\left(t\right)+ \Delta {K}_{ij}^{{L}_{2},{L}_{2}}\left(t\right)\end{array} , i\ne j \end{array}$$

14 $$\begin{array}{c}\begin{array}{c}{S}_{i}^{K2}=\sum_{j}{K}_{ij}^{{L}_{2},{L}_{2}}. \\ I\!f\; {S}_{i}^{K2}> {K}_{maxsum} \Rightarrow {K}_{ij}^{{L}_{2},{L}_{2}}\leftarrow {K}_{ij}^{{L}_{2},{L}_{2}}\cdot \frac{{K}_{maxsum} }{{S}_{i}^{K2}} \end{array} \end{array}$$

15 $$\begin{array}{c}\begin{array}{c}{\Delta A}_{ij}^{{L}_{2},{L}_{2}}\left(t\right)={\gamma }_{A}{\left({\theta }_{high2}-\frac{{z}_{f,i}^{{L}_{2}}\left(t\right)}{2{e}_{0}}\right)}^{+}{\left(\frac{{z}_{p,j}^{{L}_{2}}\left(t\right)}{2{e}_{0}}-{\theta }_{low2}\right)}^{+}\left({A}_{max}^{{L}_{2},{L}_{2}}{- A}_{ij}^{{L}_{2},{L}_{2}}\left(t\right)\right)\\ {A}_{ij}^{{L}_{2},{L}_{2}}\left(t+{T}_{s}\right)= {A}_{ij}^{{L}_{2},{L}_{2}}\left(t\right)+ \Delta {A}_{ij}^{{L}_{2},{L}_{2}}\left(t\right)\end{array} , i\ne j \end{array}$$

16 $$\begin{array}{c}\begin{array}{c}{S}_{i}^{A2}=\sum_{j}{A}_{ij}^{{L}_{2},{L}_{2}}. \\ I\!f\; {S}_{i}^{A2}> {A}_{maxsum} \Rightarrow{A}_{ij}^{{L}_{2},{L}_{2}}\leftarrow {A}_{ij}^{{L}_{2},{L}_{2}}\bullet \frac{{A}_{maxsum} }{{S}_{i}^{A2}} \end{array} \#\end{array}$$

Equations (13) and (15) have the same meaning as Eqs. (11). Equations (14) and (16) describe the same global normalization as Eq. (12).

As shown in Eq. (15), however, synapses of type A are trained with an *anti-Hebbian* rule. This signifies that the synapses are reinforced when the normalized activities in the pre-synaptic and post-synaptic populations ($\frac{{z}_{p,j}^{{L}_{2}}\left(t\right)}{2{e}_{0}}$ and $\frac{{z}_{f,i}^{{L}_{2}}\left(t\right)}{2{e}_{0}}$, respectively) are anti-correlated; in particular, the activity of pyramidal neurons in the presynaptic population, representing a stimulated object, must be above threshold, while the activity of the fast interneurons in the post-synaptic populations must below threshold, to have a synapse reinforcement.

#### Feedback synapses from layer L3 to layer L2

The training rule that we used to compute the weights of the feedback connections from L3 to L2 (17) uses a Hebb rule similar to Eqs. (12). In this case, however, we did not adopt any normalization, assuming that this does not hold for long range synapses among different layers.

17 $$\begin{array}{c}\begin{array}{c}{\Delta W}_{ij}^{{L}_{2},{L}_{3}}\left(t\right)={\gamma }_{Wb}{\left(\frac{{z}_{p,i}^{{L}_{2}}\left(t\right)}{2{e}_{0}}-{\theta }_{low3}\right)}^{+}{\left(\frac{{z}_{p,j}^{{L}_{3}}\left(t\right)}{2{e}_{0}}-{\theta }_{low3}\right)}^{+}\left({W}_{max}^{{L}_{2},{L}_{3}}{-W}_{ij}^{{L}_{2},{L}_{3}}\left(t\right)\right)\\ {W}_{ij}^{{L}_{2},{L}_{3}}\left(t+{T}_{s}\right)= {W}_{ij}^{{L}_{2},{L}_{3}}\left(t\right)+ {\Delta W}_{ij}^{{L}_{2},{L}_{3}}\left(t\right)\end{array} , i\ne j \end{array}$$

All parameters’ values are shown in Table 5.

**Table 5:** Parameters describing the training procedure

Parameter	Value
*T*_*s*_ (s)	10^–4^
$\theta$_*low*1_	0.12
$\theta$_*low*2_	0.8
$\theta$_*high*2_	0.6
$\theta$_*low*3_	0.7
${\gamma }_{W}$	0.1
${\gamma }_{K}$	1
${\gamma }_{A}$	1
${\gamma }_{Wb}$	10
${W}_{max}^{{L}_{1},{L}_{1}}$	10
${K}_{max}^{{L}_{2},{L}_{2}}$, ${K}_{max}^{{L}_{3},{L}_{3}}$	8
${A}_{max}^{{L}_{2},{L}_{2}}$, ${A}_{max}^{{L}_{3},{L}_{3}}$	0.12
${W}_{max}^{{L}_{2},{L}_{3}}$	11
${W}_{maxsum}$	130
${K}_{maxsum}$	160
${A}_{maxsum}$	$\mathrm{min}({S}_{i}^{A})$

## Code availability (software application or custom code)

The neural mass model and the programs have been implemented in Matlab. Matlab codes are available in the repository ModelDB at http://modeldb.yale.edu/267297.
